# Modulation of Allergic Sensitization and Allergic Inflammation by *Staphylococcus aureus* Enterotoxin B in an Ovalbumin Mouse Model

**DOI:** 10.3389/fimmu.2020.592186

**Published:** 2020-10-26

**Authors:** Ilka Jorde, Christina B. Hildebrand, Olivia Kershaw, Eva Lücke, Sabine Stegemann-Koniszewski, Jens Schreiber

**Affiliations:** ^1^Experimental Pneumology, Department of Pneumology, University Hospital Magdeburg/Medical Faculty, Health Campus Immunology, Infectiology and Inflammation (GC-I³), Otto-von-Guericke-University, Magdeburg, Germany; ^2^Department of Veterinary Medicine, Institute of Veterinary Pathology, Freie Universität Berlin, Berlin, Germany

**Keywords:** asthma, allergic airway inflammation, *Staphylococcus aureus*, *Staphylococcus aureus* enterotoxin B, nasal carriage, allergic sensitization

## Abstract

The superantigen *Staphylococcus aureus* (*S. aureus*) enterotoxin B (SEB) has been proposed a central player in the associations between *S. aureus* nasal colonization and the development of allergic asthma. Previously, SEB has been shown to aggravate allergic sensitization and allergic airway inflammation (AAI) in experimental mouse models. Aiming at understanding the underlying immunological mechanisms, we tested the hypothesis that intranasal (i.n.) SEB-treatment divergently modulates AAI depending on the timing and intensity of the SEB-encounter. In an ovalbumin-mediated mouse model of AAI, we treated mice i.n. with 50 ng or 500 ng SEB either together with the allergic challenge or prior to the peripheral sensitization. We observed SEB to affect different hallmark parameters of AAI depending on the timing and the dose of treatment. SEB administered i.n. together with the allergic challenge significantly modulated respiratory leukocyte accumulation, intensified lymphocyte activation and, at the higher dose, induced a strong type-1 and pro-inflammatory cytokine response and alleviated airway hyperreactivity in AAI. SEB administered i.n. prior to the allergic sensitization at the lower dose significantly boosted the specific IgE response while administration of the higher dose led to a significantly reduced recruitment of immune cells, including eosinophils, to the respiratory tract and to a significantly dampened Th-2 cytokine response without inducing a Th-1 or pro-inflammatory response. We show a remarkably versatile potential for SEB to either aggravate or alleviate different parameters of allergic sensitization and AAI. Our study thereby not only highlights the complexity of the associations between *S. aureus* and allergic asthma but possibly even points at prophylactic and therapeutic pathways.

## Introduction

Bronchial asthma is a chronic inflammatory condition affecting more than 300 million patients world-wide (1). Main symptoms are airway hyperreactivity, reversible bronchial obstruction, increased mucus production and structural changes of the airways (2). Asthma is a highly heterogeneous disease and one major discrimination is that between non-allergic and allergic asthma. With over 60%, allergic asthma represents the most frequent endotype (3). It is typically characterized by T helper type 2 cell (Th2)-dominated immune responses towards aeroallergens, including the production of allergen specific IgE antibodies, the release of Th2-inflammatory mediators such as interleukin 4 (IL-4), IL-5 and IL-13 as well as the recruitment and activation of mast cells, eosinophils, basophils and others (4, 5). Major open questions include those of inflammatory endotypes and pre-disposing factors of allergic asthma.

Today it is accepted that the airways are colonized by microorganisms that interact with the immune system in health and disease (6, 7). A significant relationship between *Staphylococcus aureus* (*S. aureus*) nasal carriage and allergic asthma has been recognized (8–12). Furthermore, colonization with *S. aureus* is associated with atopic dermatitis (13–15) and chronic rhinosinusitis (16, 17). The exact immunological interactions however remain elusive. *S. aureus* is a gram positive facultative bacterial pathogen that constantly colonizes about 30% of the adult population (18–20). Preferred sites of colonization are the skin and nasopharynx (18, 21–23). Beside its role as a commensal, *S. aureus* may induce deep skin infections and life threatening conditions such as pneumonia, sepsis and toxic shock syndrome (18, 24). Up to 80% of isolated *S. aureus* strains are capable of producing enterotoxins (12, 25–27) and especially staphylococcal enterotoxin B (SEB), typically associated with food poisoning (26, 28–30), has come into focus regarding allergic airway inflammation (AAI) (31–33). SEB belongs to the superantigen family of toxins, which are potent immune activators leading to unspecific lymphocyte activation and pro-inflammatory, mainly type 1 responses that are known to suppress Th2- and allergic responses (34–38).

So far, only few studies have experimentally addressed effects of SEB on allergic asthma and altogether suggest that SEB has a high immune-modulatory potential facilitating sensitization and aggravating allergic inflammation (31, 33, 39). A detailed knowledge of the underlying mechanisms will be essential for developing diagnostic, prophylactic and therapeutic approaches in the context of allergic asthma and nasal *S. aureus* colonization. Therefore, also in the light of epidemiological data highlighting associations between *S. aureus*, SEB and allergic asthma, we have comprehensively characterized the effects of intranasal SEB-administration on AAI in a mouse model taking further previous studies. We hypothesized that SEB would have diverging effects on AAI depending on whether it is encountered at a low or a high dose as well as before sensitization or during the allergic challenge. We show that the effects of SEB on hallmark features of AAI such as immune cell recruitment, cytokines, IgE-production, and airway hyperreactivity can be of an attenuating or intensifying nature depending on whether SEB was encountered before sensitization or during challenge and at a lower or a higher dose. As opposed to previous studies generally attesting aggravating effects of SEB on AAI, we describe SEB to ameliorate certain aspects depending on when and at which concentration it was encountered. Our study thereby adds a novel aspect to SEB-mediated modulation of AAI, underlines the profound immune-modulatory potential of SEB in the context of allergic asthma and adds important details to our understanding of this possibly clinically highly relevant interaction.

## Materials and Methods

### Mice

Female specific-pathogen free, 7–8 weeks old C57Bl/6 mice were obtained from Janvier (Saint-Berthevin, France) and housed in individually ventilated cages. All experiments were ethically reviewed, approved by the responsible authorities (Landesverwaltungsamt Sachsen-Anhalt, 203.6.3-42502-2-1495) and performed in accordance with directive 2010/63/EU.

### Intranasal SEB Treatment and Induction of Allergic Airway Inflammation

**Figure 1:**
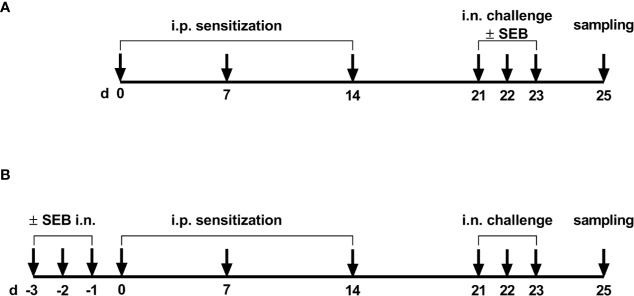
Timeline of the experimental setups. **(A)** For the induction of allergic airway inflammation (AAI), mice were sensitized three times intraperitoneally (i.p.) with 10 µg ovalbumin (OVA) and aluminum hydroxide (alum) in weekly intervals (d 0, 7, 14). One week after the last sensitization they were intranasally (i.n.) challenged with OVA in PBS on three consecutive days. For the analysis of the modulation of AAI through SEB administered with the challenge, sensitized mice were i.n. challenged with OVA alone or with OVA together with SEB (50 ng or 500 ng) on three consecutive days. **(B)** To investigate effects of i.n. SEB-treatment on allergic sensitization, mice were treated i.n. with SEB (50 ng or 500 ng) or PBS on three consecutive days, then i.p. sensitized with OVA and alum and i.n. challenged with OVA.

To study the effects of intranasal (i.n.) SEB-treatment during the allergic challenge on AAI, mice (n = 3/group) were sensitized intraperitoneally (i.p.) three times in weekly intervals with 10 µg ovalbumin (OVA; grade V, Sigma-Aldrich) in PBS containing 1 mg aluminum hydroxide (alum; Imject™ Alum Adjuvant, ThermoFisher). One week after the last sensitization, mice were i.n. challenged on three consecutive days with 100 µg OVA (grade III, Sigma-Aldrich) in 30 µl PBS (OVA/OVA) or 100 µg OVA in 30 µl PBS additionally containing 50 ng or 500 ng SEB (OVA/OVA+SEB_50_ and OVA/OVA+SEB_500_) under light isoflurane anesthesia. Analyses were performed 48 h after the last challenge (**Figure 1A**). The control group shown for these experiments was sensitized against OVA as described and challenged with PBS only (OVA/sal).

The effects of i.n. SEB-treatment before sensitization were studied by treating mice (n = 3/group) i.n. with 50 ng SEB in 30 µl PBS or 500 ng SEB in 30 µl PBS under light isoflurane anesthesia on three consecutive days prior to the first sensitization. Starting from one day after the last SEB-treatment, mice were sensitized i.p. three times in weekly intervals with 10 µg OVA in PBS containing 1 mg alum. One week after the last sensitization, mice were i.n. challenged with 100 µg OVA in 30 µl PBS under light isoflurane anesthesia on three consecutive days (SEB_50_/OVA/OVA and SEB_500_/OVA/OVA) (**Figure 1B**). The control group shown for these experiments was treated i.n. with PBS thrice prior to sensitization against OVA and was challenged with PBS only (sal/OVA/sal).

The different treatments in our models necessitated further control groups that were analyzed: (i) Mock sensitizations were performed with 1 mg aluminum hydroxide only (alum only). Mock-sensitized mice challenged with OVA (alum only/OVA) showed no features of AAI (data not shown). (ii) The acute effects of SEB alone (48 h after the last i.n. SEB-treatment) were assessed in control-immunized mice that were treated thrice i.n. with 50 ng or 500 ng SEB alone without OVA (alum only/SEB_50_ and alum only/SEB_500_). (iii) Long term effects of i.n. SEB-treatment alone (day 25) were analyzed in mice treated thrice i.n. with 50 ng SEB or 500 ng SEB before mock-sensitization and OVA challenge (SEB_50_/alum only/OVA and SEB_500_/alum only/OVA).

### Serum

Blood was collected from the retroorbital sinus and was centrifuged (4000 rpm (1500 x g), 10 min, 4 °C) after 20 min at 37 °C and 5 min at 4 °C. Serum was aliquoted and stored at -80 °C until further analysis.

### Isolation of Leukocytes

Lungs were flushed through the trachea with 1 ml ice-cold PBS for bronchoalveolar lavage (BAL). After centrifugation (2,000 rpm (360 x g), 10 min, 4 °C), the BAL supernatant was cleared from debris (10.000 x g, 5 min at 4°C) and stored at -80 °C and erythrocyte lysis was performed on the pellet. The cell pellet was used for flow-cytometric analyses. Lavaged lungs were perfused with 10 ml ice-cold PBS, excised and minced on ice followed by enzymatic digestion (45 min, 37 °C) in Iscove’s modified Dulbecco’s medium containing 0.2 mg/ml Collagenase D (Sigma-Aldrich), 0.01 mg/ml DNase (Sigma-Aldrich), and 5 % fetal calf serum. After the addition of EDTA (5 mM final concentration), suspensions were filtered (70 µm) and centrifuged (1,200 rpm (330 x g), 10 min, 4 °C). Following erythrocyte lysis by osmotic shock, leukocytes were enriched using Percoll (GE Healthcare Life Sciences). Splenocytes were isolated by homogenization of spleens through a 70 µm cell strainer, centrifugation (1,200 rpm (330 × g), 5 min, 4 °C) of the cell suspension and erythrocyte lysis by osmotic shock.

### Flow Cytometry

Cells from BAL, lung leukocytes and splenocytes were incubated with anti-CD16/CD36 (2.4G2) for blocking Fc-receptors and stained with fixable live/dead stain (BioLegend). Antibody stainings were performed for B220 (RAE6B2), CD3 (17A2), CD4 (RM4-5 or GK1.5), CD11b (M1/70), CD11c (N418), CD49b (HMα2), CD69 (H1.2F3), CD117 (2B8), FcϵRIα (MAR-1), Ly6C (HK1.4), Ly6G (1A8), MHCII (M5/114.15.2), NK1.1 (PK136) and Siglec-F (E50-2440, ThermoFisher) in different combinations (see panels below). Unless otherwise indicated, antibodies were from BioLegend. Data were acquired using an Attune NxT instrument (ThermoFisher) and analyzed using the FlowJo software (Tree Star). Single stainings were performed for all fluorochromes for compensation using UltraComp eBeads (ThermoFisher) and fluorescence-minus-one stainings were performed for gating. For the calculation of absolute cell numbers from the relative frequencies, 50,000 fluorescent beads (Precision Count Beads, BioLegend) were added to each sample. Following singlet-gating and dead cell exclusion, cell populations were gated as follows:

Leukocytes from BAL were stained with panel 1 (**Supplementary Figure 1**): Live single cells were gated for CD11c^+^, CD11b^+^/CD11c^-^ and CD11b^-^/CD11c^-^ cells. CD11c^+^ cells were further divided into macrophages (gated as CD11c^+^/Siglec-F^+^) and Siglec-F^-^ cells from which dendritic cells (DC) (CD11c^+^/Siglec-F^-^/MHCII^+^) were gated using MHCII as a marker. CD11b^+^/CD11c^-^ cells were further gated for the Ly6G and Siglec-F markers. Neutrophils were gated as CD11b^+^/CD11c^-^/Ly6G^+^ cells and eosinophils as CD11b^+^/CD11c^-^/Ly6G^-^/Siglec-F^+^ cells. CD4^+^ T cells were gated as CD11b^-^/CD11c^-^/CD4^+^ and CD8^+^ T cells as CD11b^-^/CD11c^-^/CD8^+^ cells.

Lung leukocytes and splenocytes were stained with panels 2 and 3. Panel 2 (**Supplementary Figure 2**): Live single cells were divided into CD11c^+^ and CD11c^-^ cells. The latter were further gated by using B220 and MHCII markers. B cells were gated as CD11c^-^/B220^+^/MHCII^+^ cells. The remaining CD11c^-^/B220^-^/MHCII^-^ cells were further gated for CD4^+^ T cells (CD11c^-^/B220^-^/MHCII^-^/CD4^+^) and CD8^+^ T cells (CD11c^-^/B220^-^/MHCII^-^/CD8^+^). Th2 cells were gated as ST2^+^ CD4^+^ T cells. The activation status of different cell types was determined using the CD69 marker by gating on CD69^+^ cells within the respective population.

Panel 3 (**Supplementary Figure 3**): From live single cells alveolar macrophages were gated as autofluorescence^+^ cells that were CD11b^-^/Siglec-F^+^. Autofluorescence^-^/CD3^-^/NK1.1^-^/B220^-^/CD11b^+^ cells were gated and further divided into neutrophils (CD3^-^/NK1.1^-^/B220^-^/CD11b^+^/Ly6G^+^/Siglec-F^-^) and eosinophils (CD3^-^/NK1.1^-^/B220^-^/CD11b^+^/Ly6G^-^/Siglec-F^+^). CD3^-^/NK1.1^-^/B220^-^/CD11b^+^/Ly6G^-^/Siglec-F^-^ cells were divided into inflammatory monocytes/macrophages (CD3^-^/NK1.1^-^/B220^-^/CD11b^+^/Ly6G^-^/Siglec-F^-^/Ly6C^high^) and M2-polarized macrophages (CD3^-^/NK1.1^-^/B220^-^/CD11b^+^/Ly6G^-^/Siglec-F^-^/Ly6C^low^) by using the Ly6C marker. Mast cells and basophils were gated from singlets without prior dead cell exclusion. Mast cells were gated as FcϵRIα^+^/CD117^+^/CD49^-^ and basophils as FcϵRIα^+^/CD117^+^/CD49^+^.

### Enzyme-Linked Immunosorbent Assay (ELISA)

OVA-specific IgE was detected by ELISA according to the manufacturer’s recommendations (BioLegend).

### Quantification of Cytokines in BAL

Cytokines were quantified in undiluted BAL using a 13-plex cytometric bead array according to the manufacturer’s instructions (LEGENDplex™ Th-cytokine panel, BioLegend). The following cytokines were analyzed (detection limits): IL-2 (2.22 pg/ml), IL-4 (1.34 pg/ml), IL-5 (4.07 pg/ml), IL-6 (0.69 pg/ml), IL-9 (1.22 pg/ml), IL-10 (6.65 pg/ml), IL-13 (1.70 pg/ml), IL-17A (2.14 pg/ml), IL-17F (1.85 pg/ml), IL-21 (1.72 pg/ml), IL-22 (2.15 pg/ml), IFN-γ (1.39 pg/ml), TNF-α (2.09 pg/ml).

### Assessment of Airway Hyperreactivity

Mice were anesthetized and mechanically ventilated (120 breaths/min) after tracheotomy (Buxco FinePointe R/C, DSI™ USA). After an acclimation period of 5 min, 10 µl PBS containing increasing concentrations of methacholine (0, 6.25, 12.5, 25, 25, 50, 100 mg/ml) were automatically nebulized into the breathing air (20 s delivery duration). Resistance and compliance were assessed over a 3 min response time for each methacholine concentration. After each response time there was a 1 min recovery time before nebulization of the next higher methacholine concentration. Resistance and compliance were calculated based on the single compartment lung model, using the lung pressure and air flow values that were continuously measured. For each individual animal and methacholine concentration, the average resistance over the entire respective 3 min response time was assessed. Data were analyzed using the FinePointe software.

### Histopathological Analysis

Lungs were fixed in 4 % formalin, embedded in paraffin and 5 µm sections were dewaxed and stained with hematoxylin and eosin. Blinded histological evaluations were performed by a veterinary pathologist certified by the European College of Veterinary Pathologists. The % of the tissue affected was assessed and lungs were scored (1 = mild, 2 = moderate, 3 = high) for perivascular lymphocytic infiltrates, interstitial lymphocytic infiltrates, alveolar lymphocytes, interstitial eosinophils, alveolar eosinophils, alveolar neutrophils, bronchial epithelial hyperplasia and type II pneumocyte hyperplasia. PAS (periodic-acid Schiff) staining was performed to assess accumulation of mucus and scored for goblet cell hyperplasia in the medium sized and large bronchi.

### Statistical Analysis

All statistical analyses were performed using the Graph Pad Prism software version 8 (Graph Pad Software). To assess the induction of AAI, comparison of all treatment groups to the control group was performed. To assess the effects of i.n. SEB-treatment on AAI, comparisons between all treatment groups were performed. Data for all experimental groups were tested for normality using the Shapiro-Wilk normality test. In the case of Gaussian distribution for all groups in a comparison, one-way ANOVA and Bonferroni post-hoc testing was performed. In the case of non-Gaussian distribution in at least one of the groups in a comparison, Kruskal-Wallis testing with Dunn’s post-hoc testing was performed. *P* ≤ 0.05 was considered indicative of statistical significance (*p < 0.05, ** *p* < 0.01, *** *p* < 0.005, **** *p* < 0.0001).

## Results

### Modulation of the Allergic Inflammation: SEB-Treatment During the Allergic Challenge Significantly Affects Immune Cell Recruitment to the Respiratory Tract and Alleviates Airway Hyperreactivity in AAI

**Figure 2:**
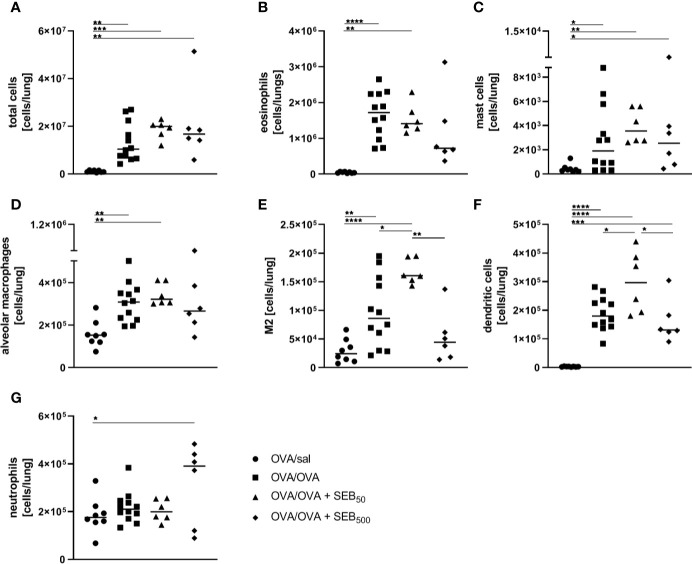
Modulation of the allergic inflammation: *S. aureus* enterotoxin B (SEB)-treatmentduring the allergen challenge leads to distinct changes in cell recruitment to the lung. For theinduction of allergic airway inflammation (AAI), mice were sensitized three times intraperitoneally(i.p.) with 10 µg ovalbumin (OVA) and alum in weekly intervals(d 0, 7, 14). One week after the last sensitization they were intranasally (i.n.)challenged with OVA (■ OVA/OVA) or OVA together with 50 ng or 500 ng SEB on three consecutivedays (50 ng SEB (▲): OVA/OVA + SEB_50_; 500 ng SEB (♦): OVA/OVA +SEB_500_). Control mice were sensitized but mock-challenged with PBS only (● OVA/sal). On day 25, lung leukocytes were analyzed for total cell counts **(A)**, absolute numbers of eosinophils **(B)**, mast cells **(C)**, alveolar macrophages **(D)**, M2-polarized monocytes/macrophages **(E)**, dendritic cells **(F)**, and neutrophils **(G)**. Data compiled from at least two independent experiments are shown for individual mice with the median. *p < 0.05, **p < 0.01, ***p < 0.005, ****p < 0.0001.

In order to assess effects of i.n. SEB-treatment on AAI, we employed an OVA-mediated mouse model. Mice were i.p. sensitized with OVA followed by the induction of AAI through an i.n. OVA-challenge. We first analyzed the influence of SEB co-administered to the airways with the allergen-challenge. We chose 50 ng SEB as a low and 500 ng SEB as a higher but sublethal dose (31, 40). As a basis for these analyses, we assessed effects of i.n. treatment with the same doses of SEB alone in mock-sensitized mice (**Supplementary Figure 4**). Following treatment with 500 ng SEB we observed a significant increase in total cell numbers, CD4^+^ T cell numbers and eosinophils in the lungs and BAL. Also respiratory levels of IFN-γ, IL-5 and TNF-α were significantly increased. Treatment with 50 ng SEB did not lead to significant changes in these parameters (**Supplementary Figure 4A-I**). For a detailed characterization of the effects of SEB-treatment on AAI, mice were treated with SEB together with the allergic challenge (**Figure 1A**). In the lungs, induction of AAI alone led to a significant increase in total cell numbers, eosinophils, mast cells, alveolar macrophages, M2-polarized monocytes/macrophages and DC (**Figures 2A–G**). Also the frequency of M2-polarized monocytes/macrophages within the monocyte/macrophage population was significantly increased following the induction of AAI (data not shown). The total lung leukocyte number was not significantly altered by additional intranasal treatment with 50 ng or 500 ng SEB during the allergic challenge (**Figure 2A**). Nevertheless, treatment with 50 ng SEB led to significantly increased numbers (but not frequency; data not shown) of M2-polarized monocytes/macrophages and DC as compared to AAI alone (**Figures 2E, F**). Treatment with 500 ng SEB led to rather reduced numbers of these cells as compared to AAI alone and to significantly reduced numbers as compared to treatment with 50 ng SEB (**Figures 2E, F**). Lung neutrophils were not significantly elevated in AAI alone but in AAI combined with the treatment with 500 ng SEB during the challenge (**Figure 2G**).

**Figure 3:**
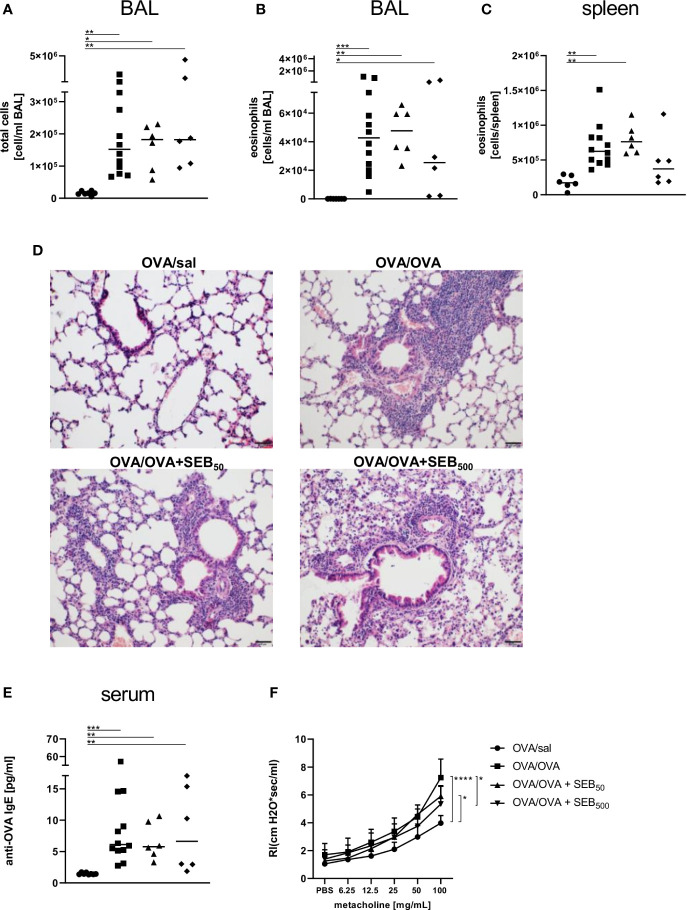
Modulation of the allergic inflammation: *S. aureus* enterotoxin B (SEB)-treatmentduring the allergen challenge significantly affects airway hyperreactivity. Ovalbumin(OVA)-sensitized mice were i.n. challenged with OVA (■ OVA/OVA) or OVA together with 50 ng or500 ng SEB (50 ng SEB (▲): OVA/OVA + SEB_50_; 500 ng SEB (♦): OVA/OVA +SEB_500_). Control mice were mock-challenged with PBS only (● OVA/sal). On day 25, bronchoalveolar lavage (BAL) leukocytes were analyzed regarding the total cell count **(A)** and eosinophil numbers **(B)** and splenocytes were analyzed for eosinophil numbers **(C)**. Lungs (n = 4 controls, n = 5 in treatment groups) were stained with hematoxylin and eosin, scale bar = 50 µm **(D)**. OVA-specific IgE in the serum **(E)** and airway hyperreactivity (n=6/group from two independent experiments) were assessed **(F)**. In **(A–C, E)** data compiled from at least two independent experiments and the median are shown for individual mice. *p < 0.05, **p < 0.01, ***p < 0.005. In **(D)** representative images are shown. In **(F)** data are shown as mean + SD. *P*-values (*p < 0.05, ****p < 0.0001) refer to RI after 100 mg/ml methacholine.

In the BAL, the induction of AAI led to a significant increase of total cell numbers and eosinophils (**Figures 3A, B**) and eosinophil numbers were also significantly elevated in the spleen following induction of AAI alone (**Figure 3C**). Treatment with 50 ng or 500 ng SEB during the allergic challenge did not significantly affect these parameters as compared to AAI alone (**Figures 3A–C**). There was however no significant elevation in splenic eosinophils in AAI, if mice were treated with 500 ng SEB during the allergic challenge (**Figure 3C**). In AAI, lung lesions were histologically characterized by typical bronchointerstitial pneumonia with a strong involvement of eosinophils. Mild bronchial epithelial hyperplasia and pneumocyte type II hyperplasia were observed. After treatment with either 50 ng or 500 ng SEB during the allergic challenge, these changes were similar (**Figure 3D**), however with milder perivascular lymphocytic infiltrates but moderate interstitial lymphocytic infiltrates and moderate type II pneumocyte hyperplasia. Accumulation of mucus was observed only sporadically but goblet cell hyperplasia in the medium sized and predominantly large bronchi was present in AAI and was not affected by SEB-treatment during the challenge (**Supplementary Figure 5**). Compared to unchallenged controls, induction of AAI alone led to significantly increased levels of OVA-specific IgE antibodies which were not altered by additional treatment with either 50 ng or 500 ng SEB (**Figure 3E**). Airway hyperreactivity, assessed as airway resistance in response to methacholine, was significantly elevated in AAI alone and in AAI combined with the i.n. treatment with 50 ng SEB, but not in AAI combined with the i.n. treatment with 500 ng SEB during the allergic challenge (**Figure 3F**). Treatment with 500 ng SEB during the allergic challenge resulted in significantly decreased airway hyperreactivity as compared to AAI alone (**Figure 3F**).

In summary, treatment with 50 ng SEB during the allergic challenge significantly increased M2-polarized monocytes/macrophages and DC in the lungs without significantly affecting airway hyperreactivity as compared to AAI alone. In contrast, likewise treatment with 500 ng SEB significantly reduced absolute numbers of M2-polarized monocytes/macrophages and DC as compared to treatment with 50 ng SEB while at the same time attenuating airway hyperreactivity as compared to AAI alone.

### Modulation of the Allergic Inflammation: SEB-Treatment During the Allergic Challenge Significantly Affects Lymphocyte Activation and Cytokines in the Respiratory Tract in AAI

**Figure 4:**
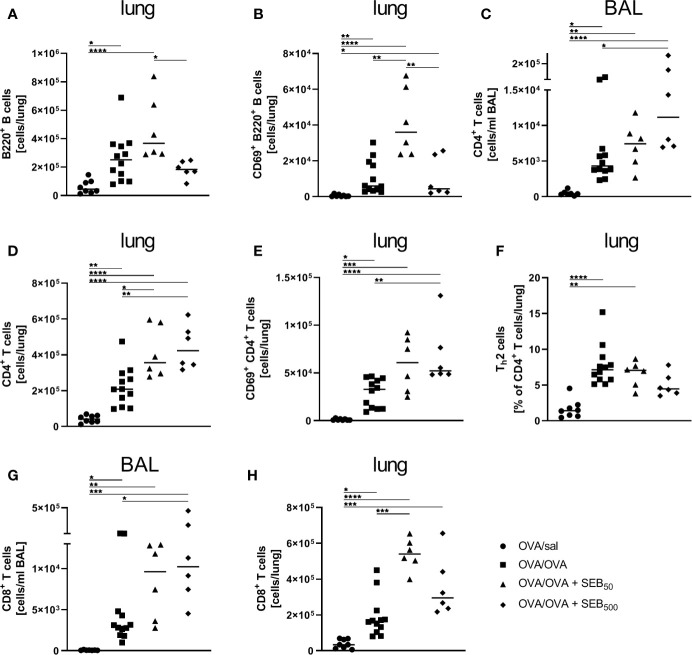
Modulation of the allergic inflammation: *S. aureus* enterotoxin B (SEB)-treatmentduring the allergen challenge leads to increased numbers of activated lung lymphocytes. Ovalbumin(OVA)-sensitized mice were i.n. challenged with OVA (■ OVA/OVA) or OVA together with 50 ng or500 ng SEB (50 ng SEB (▲): OVA/OVA + SEB_50_; 500 ng SEB (♦):OVA/OVA + SEB_500_). Control mice were mock-challenged with PBS only (● OVA/sal). On day 25, lungs and bronchoalveolar lavage (BAL) were analyzed for the absolute numbers of B220^+^ B cells **(A)** and CD69^+^ B220^+^ B cells **(B)** in the lungs, CD4^+^ T cells (BAL **(C)**, lung **(D)**), CD69^+^ CD4^+^ T cells (lung **(E)**), the frequency of Th2 cells within CD4^+^ T cells (lung **(F)**) and CD8^+^ T cells (BAL **(G)**, lung **(H)**). Data compiled from at least two independent experiments are shown for individual mice with the median. *p < 0.05, **p < 0.01, ***p < 0.005, ****p < 0.0001.

To characterize the effects of i.n. SEB-treatment during allergen encounter on the phenotype of AAI in more detail, we assessed lymphocyte numbers and lymphocyte expression of the activation marker CD69 as well as cytokine levels in the respiratory tract of mice treated with SEB together with the allergic challenge. The induction of AAI alone led to significantly elevated numbers of B220^+^ B cells and CD69^+^ B220^+^ B cells in the lungs (**Figures 4A, B**). CD4^+^ and CD8^+^ T cells were significantly increased in the BAL (**Figures 4C, G**) and lungs (**Figures 4D, H**) and CD69^+^ CD4^+^ T cells were significantly increased in the lungs (**Figure 4E**). Also the frequency of Th2 cells in the lungs was significantly elevated in AAI alone (**Figure 4F**). Treatment with 50 ng SEB during the allergic challenge did not affect total B220^+^ B cell numbers in the lungs (**Figure 4A**) but led to a significant increase of CD69-expressing B220^+^ B cells as compared to AAI alone (**Figure 4B**). Furthermore, total CD4^+^ T cell and CD8^+^ T cell numbers (**Figures 4D, H**) in the lungs were significantly elevated as compared to AAI alone. Treatment with 500 ng SEB during the allergic challenge did not affect B cell numbers or activation in the lungs as compared to AAI alone (**Figures 4A, B**). Nevertheless, total CD4^+^ in the BAL and lungs (**Figures 4C, D**) and CD8^+^ T cells in the BAL (**Figure 4G**) and activated CD4^+^ T cells in the lungs (**Figure 4E**) were significantly increased as compared to AAI alone. In contrast, while in AAI alone and in AAI in combination with 50 ng SEB there was a significant increase in the frequency of Th2 cells in the lungs (**Figure 4F**), this was not the case in AAI in combination with 500 ng SEB administered during the allergic challenge (**Figure 4F**).

Taken together, CD4^+^ T cell numbers in the lung were likewise affected by treatment with 50 ng and 500 ng SEB. Furthermore, treatment with 50 ng SEB explicitly intensified B cell activation and CD8^+^ T cell numbers in the lung, while treatment with 500 ng SEB significantly increased CD8^+^ T cell numbers in the BAL, intensified CD4^+^ T cell activation in the lung and interfered with Th2 cell recruitment/polarization in the lungs in AAI.

**Figure 5:**
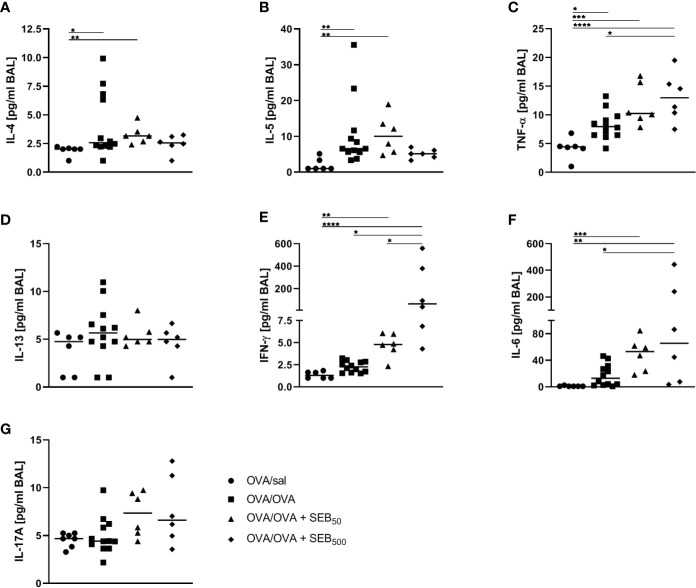
Modulation of the allergic inflammation: *S. aureus* enterotoxin B (SEB)-treatmentduring the allergic challenge leads to an increased production of pro-inflammatory cytokines in therespiratory tract. Ovalbumin (OVA)-sensitized mice were i.n. challenged with OVA (■ OVA/OVA)or OVA together with 50 ng or 500 ng SEB (50 ng SEB (▲): OVA/OVA + SEB_50_;500 ng SEB (ɦ): OVA/OVA + SEB_500_). Control mice were mock-challenged with PBSonly (● OVA/sal). On day 25, bronchoalveolar lavage (BAL) was analyzed for the concentrations of IL-4 **(A)**, IL-5 **(B)**, TNF-α **(C)**, IL-13 **(D)**, IFN-γ **(E)**, IL-6 **(F)**, and IL-17A **(G)**. Data compiled from at least two independent experiments are shown for individual mice with the median. *p < 0.05, **p < 0.01, ***p < 0.005, ****p < 0.0001.

AAI is typically accompanied by the local production of characteristic cytokines such as IL-4 and IL-5 as well as pro-inflammatory mediators. On the basis of the significant SEB-mediated changes we observed in the immune cell composition and lymphocyte activation in the airways in AAI, we assessed the respiratory cytokine profile following the induction of AAI with and without additional SEB-treatment during the allergic challenge. AAI was characterized by a significant increase in IL-4, IL-5 and TNF-α in the BAL (**Figures 5A–C**). There was no significant induction of IL-13, IFN-γ, IL-6 or IL-17A (**Figures 5D–G**). Treatment with 50 ng SEB during the allergic challenge did not significantly affect levels of any of these cytokines. Treatment with 500 ng SEB during the allergic challenge had no significant effect on the concentrations of IL-4, IL-5, IL-13 and IL-17 in the BAL (**Figures 5A, B, D, G**), while at the same time IL-4 and IL-5 were not significantly increased as compared to the control, which was however the case for AAI alone and AAI in combination with 50 ng SEB (**Figures 5 A, B**). Furthermore there were significantly increased concentrations of TNF-α, IFN-γ and IL-6 as compared to AAI alone (**Figures 5C, E, F**).

Taken together, treatment with 500 ng SEB during the allergic challenge as compared to AAI alone led to increased airway IFN-γ, IL-6 and TNF-α, while dampening the AAI-associated induction of Th2 cytokines. These effects on the cytokine profile pointed at a possible shift towards a rather Th1- and pro-inflammatory cytokine response.

### Modulation of the Allergic Sensitization: SEB-Treatment Prior to Sensitization Significantly Affects Immune Cell Recruitment and IgE-Production in AAI

**Figure 6:**
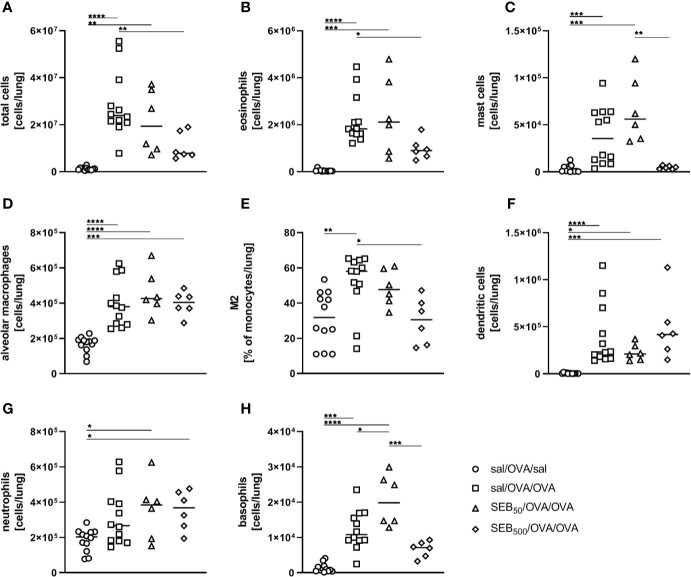
Modulation of sensitization: *S. aureus* enterotoxin B (SEB)-treatment prior tothe first sensitization leads to distinct changes in the recruitment of cells to the respiratorytract in allergic airway inflammation (AAI). Mice were treated i.n. with 50 ng or 500 ng SEB or PBS(control; sal) on three consecutive days, then i.p. sensitized with ovalbumin (OVA) and alum andi.n. challenged with OVA (50 ng SEB (Δ): SEB_50_/OVA/OVA; 500 ng SEB(◊): SEB_500_/OVA/OVA; AAI (□): sal/OVA/OVA). Control mice were mockchallenged i.n. with PBS only (○ sal/OVA/sal). On day 25, lung leukocytes were analyzed for the total cell count **(A)**, absolute numbers of eosinophils **(B)**, mast cells **(C)**, alveolar macrophages **(D)**, the frequency of M2-polarized monocytes/macrophages **(E)**, absolute numbers of dendritic cells **(F)**, neutrophils **(G)**, and basophils **(H)**. Data compiled from at least two independent experiments are shown for individual mice with the median. *p < 0.05, **p < 0.01, ***p < 0.005, ****p < 0.0001.

Based on the effects on AAI we observed for SEB administered together with the allergic challenge (summarized in **Table 1**), we asked whether SEB administered i.n. before the peripheral sensitization also has the potential to modulate AAI. To address possible effects of SEB-treatment before sensitization, mice were i.n. treated with SEB on three consecutive days before the first i.p. sensitization (**Figure 1B**). Long-term effects of intranasal SEB-treatment alone were assessed in unsensitized control animals (day 25). Here, mice treated with 50 ng and 500 ng SEB without the induction of AAI still showed significantly increased total cell numbers in the lung (**Supplementary Figure 6A**). Total cell counts in the BAL as well as CD4^+^ T cell and eosinophil numbers in lungs and BAL were not significantly affected (**Supplementary Figure 6B-F**). Furthermore, at this time-point we did not any longer detect significant changes in the levels of IFN-γ, IL-5 or TNF-α in the respiratory tract (**Supplementary Figure 6G-I**). I.n. treatment with 50 ng SEB before the first sensitization did not affect total cell numbers, eosinophils, mast cells, alveolar macrophages, the frequency (or total number, data not shown) of M2-polarized monocytes/macrophages, DC or neutrophils as compared to AAI alone (**Figures 6A–G**). However, the total number of basophils in the lungs was significantly increased as compared to AAI alone (**Figure 6H**). I.n. pre-treatment with 500 ng SEB before the first sensitization led to significantly reduced total cell numbers in the lungs as compared to AAI alone (**Figure 6A**). There were no changes in the absolute numbers of mast cells, alveolar macrophages, DC, neutrophils or basophils (**Figures 6C, D, F–H**). However, as compared to AAI alone, treatment with 500 ng SEB before sensitization led to significantly reduced numbers of eosinophils and a reduced frequency (but not absolute numbers, data not shown) of M2-polarized monocytes/macrophages in the lungs (**Figures 6B, E**).

**Table 1:** Summary of the significant effects of i.n. *S. aureus* enterotoxin B (SEB)-treatment on allergic airway inflammation (AAI).

i.n. SEB-treatment	SEB-dose	AAI parameter	Effect
with challenge	50 ng	cells in respiratory tract	
		lung M2 macrophages/monocytes	increased
		lung DC	increased
		lung CD69^+^ B220^+^ B cells	increased
		lung CD4^+^ T cells	increased
		lung CD8^+^ T cells	increased
	500 ng	airway hyperreactivity	
		response to metacholine	decreased
		cells in respiratory tract	
		BAL CD4^+^ T cells	increased
		lung CD4^+^ T cells	increased
		lung CD69^+^ CD4^+^ T cells	increased
		BAL CD8^+^ T cells	increased
		respiratory cytokines	
		BAL IFN-γ	increased
		BAL IL-6	increased
		BAL TNF-α	increased
prior to sensitization	50 ng	IgE response	
		serum OVA-specific IgE	increased
		cells in respiratory tract	
		lung basophils	increased
	500 ng	cells in respiratory tract	
		lung total cells	decreased
		BAL total cells	decreased
		lung eosinophils	decreased
		lung M2 macrophage/monocyte frequency	decreased
		respiratory cytokines	
		IL-4	decreased
		IL-5	decreased
		IL-6	decreased
		IL-17A	decreased

The table lists significant SEB-mediated effects detected on parameters of AAI as described in the Results section. All significant changes for the respective SEB-treated groups as compared to AAI alone are listed.

**Figure 7:**
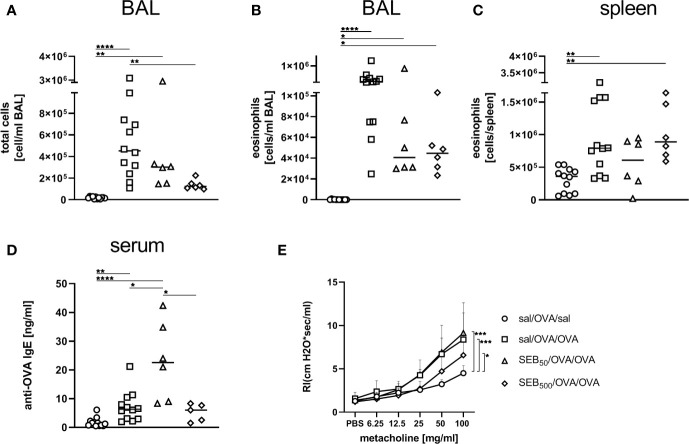
Modulation of sensitization: *S. aureus* enterotoxin B (SEB)-treatment prior tothe first sensitization leads to changes in bronchoalveolar lavage (BAL) leukocyte numbers and serumIgE in allergic airway inflammation (AAI). Mice were treated i.n. with 50 ng or 500 ng SEB or PBS(control; sal) on three consecutive days, then i.p. sensitized with ovalbumin (OVA) and alum andi.n. challenged with OVA (50 ng SEB (Δ): SEB_50_/OVA/OVA; 500 ng SEB(◊): SEB_500_/OVA/OVA; AAI (□): sal/OVA/OVA). Control mice weremock-challenged i.n. with PBS only (o sal/OVA/sal). On day 25, leukocytes from BAL were analyzed for the total cell count **(A)** and absolute number of eosinophils **(B)**. Splenocytes were analyzed for eosinophils **(C)**. OVA-specific IgE-antibodies were assessed in the serum **(D)** and airway hyperreactivity (n=6/group from two experiments) was analyzed **(E)**. In **(A–C)**, and **(D)** data compiled from at least two independent experiments and the median are shown for individual mice. *p < 0.05, **p < 0.01, ****p < 0.0001. In **(E)** data are shown as mean + SD. *P*-values (*p < 0.05, ***p < 0.005) refer to RI after 100 mg/ml, methacholine.

Also in the BAL, total cell counts and eosinophil numbers were unchanged between AAI alone and pre-treatment with 50 ng SEB (**Figures 7A, B**). Treatment with 500 ng SEB before sensitization however led to a significant reduction in total cell number in BAL (**Figure 7A**), as had also been observed for the lungs (**Figure 6A**). Eosinophil numbers in the BAL and spleen were not significantly altered between AAI alone and treatment with 50 ng or 500 ng SEB before sensitization (**Figures 7B, C**). There was a clear and significant increase in the production of OVA-specific IgE antibodies in mice i.n. treated with 50 ng SEB before sensitization as compared to AAI alone (**Figure 7D**). At the same time, i.n. SEB-treatment before sensitization at either dose, in contrast to treatment together with the challenge, did not significantly affect airway hyperreactivity in AAI (**Figures 7E**).

In summary, as compared to AAI alone, i.n. treatment with 50 ng SEB before sensitization led to a significant increase of basophils in the lungs and to significantly increased serum OVA-specific IgE antibody levels. In contrast, likewise treatment with 500 ng SEB led to significantly decreased numbers of eosinophils as well as a significantly reduced frequency of M2-polarized monocytes/macrophages in the lungs. These data (summarized in **Table 1**) clearly show a long-term potential for intranasal SEB to modulate AAI, possibly also through affecting peripheral allergic sensitization.

### Modulation of the Allergic Sensitization: SEB-Treatment Before Sensitization Does Not Affect Respiratory Lymphocyte Activation but Cytokine Production in AAI

**Figure 8:**
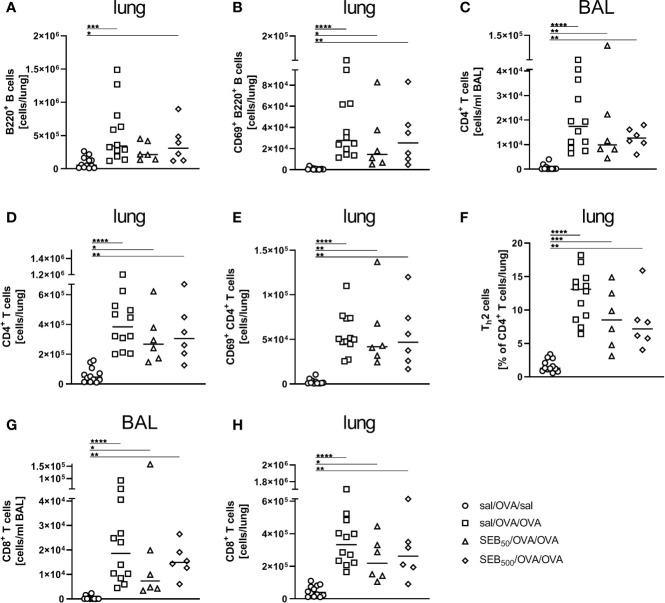
Modulation of sensitization: Intranasal treatment with *S. aureus* enterotoxin B(SEB) prior to the first sensitization does not affect respiratory lymphocyte activation in allergicairway inflammation (AAI). Mice were treated i.n. with 50 ng or 500 ng SEB or PBS(control; sal) on three consecutive days, then i.p. sensitized with ovalbumin (OVA) and alum andi.n. challenged with OVA (50 ng SEB (Δ): SEB_50_/OVA/OVA; 500 ng SEB(◊): SEB_500_/OVA/OVA; AAI (□): sal/OVA/OVA). Control mice weremock-challenged i.n. with PBS only (○ sal/OVA/sal). On day 25, lungs and bronchoalveolar lavage (BAL) were analyzed for the absolute numbers of B220^+^ B cells (lung **(A)**) and B220^+^ CD69^+^ B cells [lung **(B)**], CD4^+^ T cells [BAL **(C)**, lung **(D)**], CD69^+^ CD4^+^ T cells [lung **(E)**], the frequency of Th2 cells of CD4^+^ T cells [lung **(F)**] and CD8^+^ T cells (BAL **(G)**, lung **(H)**) Data compiled from at least two independent experiments are shown for individual mice together with the median. *p < 0.05, **p < 0.01, ***p < 0.005, ****p < 0.0001.

**Figure 9:**
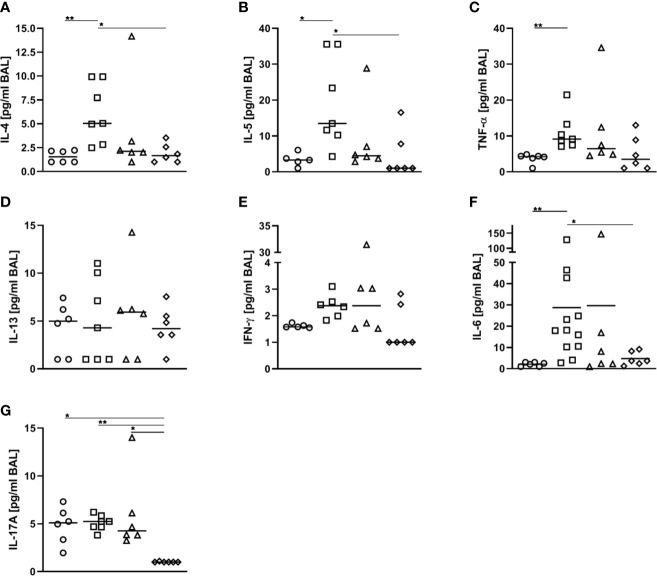
Modulation of sensitization: Treatment with 500 ng *S. aureus* enterotoxin B(SEB) prior to sensitization leads to decreased type-2 cytokines in allergic airway inflammation(AAI). Mice were treated i.n. with 50 ng or 500 ng SEB or PBS (control; sal) on threeconsecutive days, then i.p. sensitized with ovalbumin (OVA) and alum and i.n. challenged with OVA(50 ng SEB (Δ): SEB_50_/OVA/OVA; 500 ng SEB (◊):SEB_500_/OVA/OVA; AAI (□): sal/OVA/OVA). Control mice were mock-challenged i.n. withPBS only (○ sal/OVA/sal). On day 25, bronchoalveolar lavage (BAL) was analyzed for IL-4 **(A)**, IL-5 **(B)**, TNF-α **(C)**, IL-13 **(D)**, IFN-γ **(E)**, IL-6 **(F)**, and IL-17A **(G)**. Data from two independent experiments are shown for individual mice with the median. *p < 0.05, **p < 0.01.

As compared to AAI alone, neither pre-treatment with 50 ng or 500 ng SEB affected the number of total and CD69-expressing B220^+^ B cells and CD4^+^ T cells, the total number of CD8^+^ T cells or the frequency of Th2 CD4^+^ T cells in the lungs (**Figures 8A, B, D–F, H**). Also BAL CD4^+^ and CD8^+^ T cells were not affected (**Figures 8C, G**). With regard to respiratory cytokines, pre-treatment with 50 ng SEB did not significantly affect IL-4, IL-5, TNF-α, IL-13, IFN-γ, IL-6 or IL-17A (**Figures 9A–G**). As compared to the controls, TNF-α was significantly induced only in AAI alone but not in AAI after treatment with 50 ng or 500 ng SEB (**Figure 9C**). Treatment with 500 ng SEB before sensitization led to significantly reduced levels of IL-4, IL-5, IL-6 and IL-17A as compared to AAI alone (**Figures 9A, B, F, G**).

In conclusion, SEB i.n. administered before peripheral sensitization, next to affecting recruitment of major effector cells (50 ng and 500 ng) and the production of allergen-specific IgE antibodies (50 ng), profoundly modulated Th2 and pro-inflammatory respiratory cytokine production (500 ng) in AAI.

## Discussion

*S. aureus* is one of the most important bacterial pathogens. It is widely accepted that next to frequent persistent colonization every individual gets into contact with it at least once in their life (18, 41–43). Furthermore, it has been shown that the skin of over 90% of patients with atopic dermatitis is colonized by *S. aureus* and that disease severity directly correlates to biofilm growth (13, 44–46). Also patients with chronic rhinosinusitis are frequently colonized by *S. aureus* (16, 47) and a significant relationship between nasal *S. aureus* carriage and asthma severity has been recognized (8).

Different *S. aureus* proteins, such as, e.g., serine protease like proteases (Spls), have come into focus regarding Th2-biased immune responses (48) and also *S. aureus* toxins have been proposed to contribute to the development of allergic diseases in clinical and experimental studies (49, 50). Clinical studies analyzing *S. aureus* isolates recovered from nasal carriers showed occurrence of SEB-producing strains (51, 52), whereas to our knowledge reports on natural concentrations of SEB produced by *S. aureus* in the human respiratory tract are lacking. Different groups have performed dosing studies for i.n. SEB-treatment in naïve and sensitized wild-type mice with respect to parameters such as the recruitment of leukocytes to the respiratory tract and have observed significant reactions following treatment with 50 ng and 500 ng SEB, without signs of wasting disease (31, 40). In line with these studies, we did not observe significant effects following i.n. treatment with 5 ng SEB alone (data not shown) and based on the previous reports and our results we chose to analyze i.n. treatment with 50 ng and 500 ng SEB during the allergic challenge and before sensitization. Few previous experimental studies have performed treatment of wild-type mice with SEB in different models of AAI and demonstrated a strong immune modulatory potential (31, 33, 39). Combined epicutaneous SEB/OVA-sensitization before airway OVA-challenge led to increased inflammatory cells in the lungs as compared to mice sensitized with OVA alone (39). Furthermore i.n. SEB-treatment, and not any other *S. aureus* toxin, together with OVA facilitated respiratory sensitization and resulted in increased serum levels of OVA-specific IgE, a significant recruitment of eosinophils and lymphocytes and increased airway hyperreactivity (33). In a similar approach to ours, peripherally OVA-sensitized mice were treated i.n. with 500 ng SEB before aerosol challenge. Here, SEB led to increased cell counts and especially eosinophils in BAL-cytospins and to enhanced bronchial mRNA levels for IL-5, IL-4, eotaxin-1, IL-12 p40, IFN-γ and TGF-β (31). We aimed at taking these important observations further towards unraveling the underlying immunological mechanisms, especially as also the clinical associations between SEB and allergic asthma remain mechanistically unclear.

We observed that treatment with 50 ng SEB during the allergic challenge led to a significant increase of DC and M2-ploarized monocytes/macrophages in the lungs. While Muraille et al. observed that intravenous injection of SEB led to decreased DC numbers in the spleen (53), Yoon et al. demonstrated that i.p.-injected SEB is a potent activator of splenic DC (54). Krysko et al. treated mice i.n. with OVA, SEB or a combination of both and observed increased numbers of DC of various phenotypes following combined treatment (55). Our data are in line with the recent observation that *S. aureus* induces type-2 polarization of monocytes/macrophages and the activation of M2-polarized macrophages through the production of enterotoxins. Such polarized macrophages show decreased phagocytosis of *S. aureus* allowing its long term survival (12, 56). Also, macrophages are potent modulators of asthma (57) and experimental studies showed that the inhibition of M2-polarization aggravates airway hyperreactivity (58) while vice versa airway hyperreactivity can be alleviated by increased M2 polarization (59). As opposed to these findings, we observed that airway hyperreactivity was not significantly affected despite significantly increased numbers of M2 macrophages in mice treated with 50 ng SEB during the allergic challenge. On the other hand, treatment with 500 ng SEB together with the allergic challenge led to significantly alleviated airway hyperreactivity while M2-polarization was not significantly altered as compared to AAI alone. In treatment with 500 ng SEB before sensitization the frequency of M2-polarized macrophages was significantly less as compared to AAI alone, while at the same time airway hyperreactivity was also not significantly affected. As development of airway hyperreactivity itself is a multifactorial process (60), one can only speculate that in our models alternative mechanisms overlaying the expected effects of altered M2 polarization on airway hyperreactivity are at play. Possibly also the sex of the mice used in our experiments plays a role, as generally airway hyperreactivity in response to methacholine is less pronounced in female mice (61, 62). With respect to the superantigen properties of SEB (28, 63, 64) and related effects on lymphocytes, we next to a dose-dependent increase of CD4^+^ and CD8^+^ T cell numbers and CD69-expression in the respiratory tract show significantly increased numbers of CD69-expressing B cells in the lungs after i.n. treatment with 50 ng SEB during the allergic challenge. This is possibly a direct effect of SEB on respiratory B cells, as staphylococcal enterotoxins are potent B cell activators in human PBMC cultures (65, 66). Furthermore, treatment with 500 ng SEB during the allergic challenge led to an increase of pro-inflammatory cytokines and to significantly increased concentrations of IFN-γ, which mirrors the typical antibacterial type-1 immune response after *S. aureus* infection (28, 67, 68). While other reports showed increased levels of typical type-2 cytokines after i.n. treatment with SEB alone or in combination with OVA (31, 33, 37, 40, 69), we observed no significant changes in IL-4, IL-5 and IL-13 in AAI, if mice had been treated with SEB during the allergic challenge. Our observations suggest that the SEB-induced Th1- and pro-inflammatory cytokine response possibly shifts the balance away from, or at least does not generally boost, the allergy-associated Th2-response (70). This is further supported by the reduced frequency of Th2 cells and the reduced number of M2-polarized macrophages we observed in the lungs of mice treated with 500 ng SEB during the allergic challenge, as both the frequency of Th2 cells and the number of M2 macrophages in the lung were significantly elevated in AAI alone, but not in AAI in combination with 500 ng SEB during the allergic challenge. These changes were associated with significantly decreased airway hyperreactivity, showing that SEB-mediated modulation of AAI can have beneficial effects on lung functional parameters. This finding is remarkable, as generally SEB is associated with an aggravation of AAI (31, 71).

Intraperitoneal injection of SEB leads to lung inflammation (72) and intranasal SEB facilitates respiratory sensitization (33). However, the question whether i.n. SEB-treatment would affect peripheral sensitization has not been addressed. Administration of SEB prior to sensitization indeed showed distinct effects on subsequently induced AAI. Administration of 50 ng SEB prior to sensitization led to significantly elevated levels of OVA-specific IgE in the serum, suggesting that SEB encountered *via* the respiratory tract has the potential to enhance peripheral allergic sensitization. At this point it remains elusive, how this effect is mediated. Possibly, dissemination of local inflammatory mediators to the periphery or even neuronal pathways play a role. Clinical studies have shown that *S. aureus* colonization in atopic dermatitis leads to a higher degree of allergic sensitization and a higher frequency of asthma (73). Furthermore a role for SEB and also for SEB-specific IgE-antibodies in allergic sensitization and in asthma pathogenesis has been proposed (74, 75). Treatment with 500 ng SEB prior to sensitization affected AAI at multiple levels resulting in altogether ameliorated allergic inflammation. Future studies will have to address whether this finding reflects acute effects on sensitization, long-term SEB-mediated effects on local and peripheral lymphocytes, on the microenvironment of the respiratory tract including the respiratory epithelium or a combination of these. Then, a central question will be whether these effects can in any way be exploited for therapeutic purposes.

Ultimately, our study demonstrates that the potential of SEB to modulate AAI is exceptionally versatile and at different levels can affect allergic sensitization, inflammation as well as airway hyperreactivity (**Table 1**). While administration of 500 ng SEB during the allergic challenge shifted inflammation towards a Th1- and pro-inflammatory phenotype and ameliorated airway hyperreactivity, administration of the same dose of SEB prior to sensitization generally dampened AAI without significantly enhancing Th1- or pro-inflammatory parameters. We describe for the first time that i.n. SEB-treatment before peripheral sensitization has a clear potential to boost the allergen-specific IgE-response. As opposed to previous reports, we therefore propose SEB rather to specifically modulate than to generally aggravate allergic processes, which in the future could potentially even open therapeutic pathways. Our major finding is that SEB-mediated modulation of AAI can have beneficial or detrimental consequences that strongly depend on whether SEB is encountered before allergic sensitization or concurrent with the allergic challenge and on the intensity of this encounter. Future experimental and clinical studies will have to disentangle the exact interplay between SEB, allergic sensitization and airway inflammation depending on when, where and also on how intensely SEB is encountered. Such studies will have to elucidate which of the SEB-mediated effects relate to the superantigen-activity of SEB on lymphocytes and which are mediated by lymphocyte-independent pathways such as modulation of respiratory epithelial cell responses or direct skewing of the phenotype and function of alveolar macrophages and monocytes. Our observations raise the central question, which SEB-mediated changes ultimately tip the immunological balance aggravating or alleviating AAI. Such knowledge will enable targeted strategies of prevention and therapy, especially in the light of *S. aureus* infections and colonization as well as of the large number of *S. aureus* carriers and patients suffering from allergic asthma. Such measures could possibly range from screening for toxigenic *S. aureus* carriage, targeted de-colonization or targeting of suppressive pathways in inflammation and airway hyperreactivity.

## Data Availability Statement

The raw data supporting the conclusions of this article will be made available by the authors, without undue reservation.

## Ethics Statement

The animal study was reviewed and approved by Landesverwaltungsamt Sachsen-Anhalt.

## Author Contributions

IJ planned experiments, performed experiments, analyzed the data and wrote the manuscript. CBH and OK performed experiments and analyzed the data. EL analyzed data. SS-K planned and supervised the study, planned and performed experiments, analyzed data and wrote the manuscript. JS planned and supervised the study and wrote the manuscript. All authors contributed to the article and approved the submitted version.

## Funding

IJ is supported by the German Research Foundation through grant 361210922/RTG 2408.

## Conflict of Interest

The authors declare that the research was conducted in the absence of any commercial or financial relationships that could be construed as a potential conflict of interest.
