# Supplementary material for: Modulation of Allergic Sensitization and Allergic Inflammation by Staphylococcus aureus Enterotoxin B in an Ovalbumin Mouse Model
Source: Front Immunol. 2020 Oct 26;11:592186. doi: 10.3389/fimmu.2020.592186 (PMC7649385; doi:10.3389/fimmu.2020.592186)
Supplement: Supplementary file 1 [file DataSheet_1.pdf]

Supplementary Material

(A)

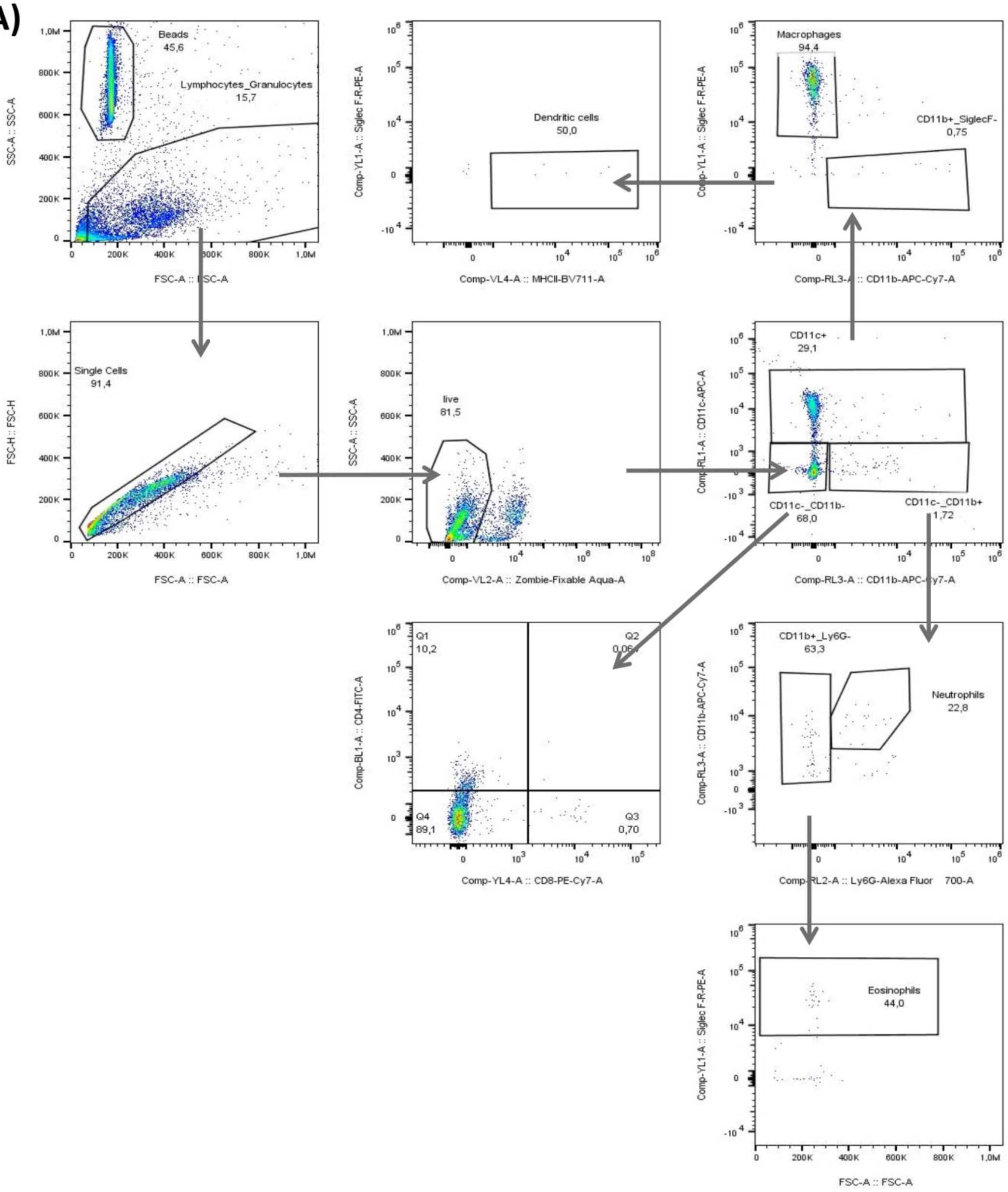

(B)

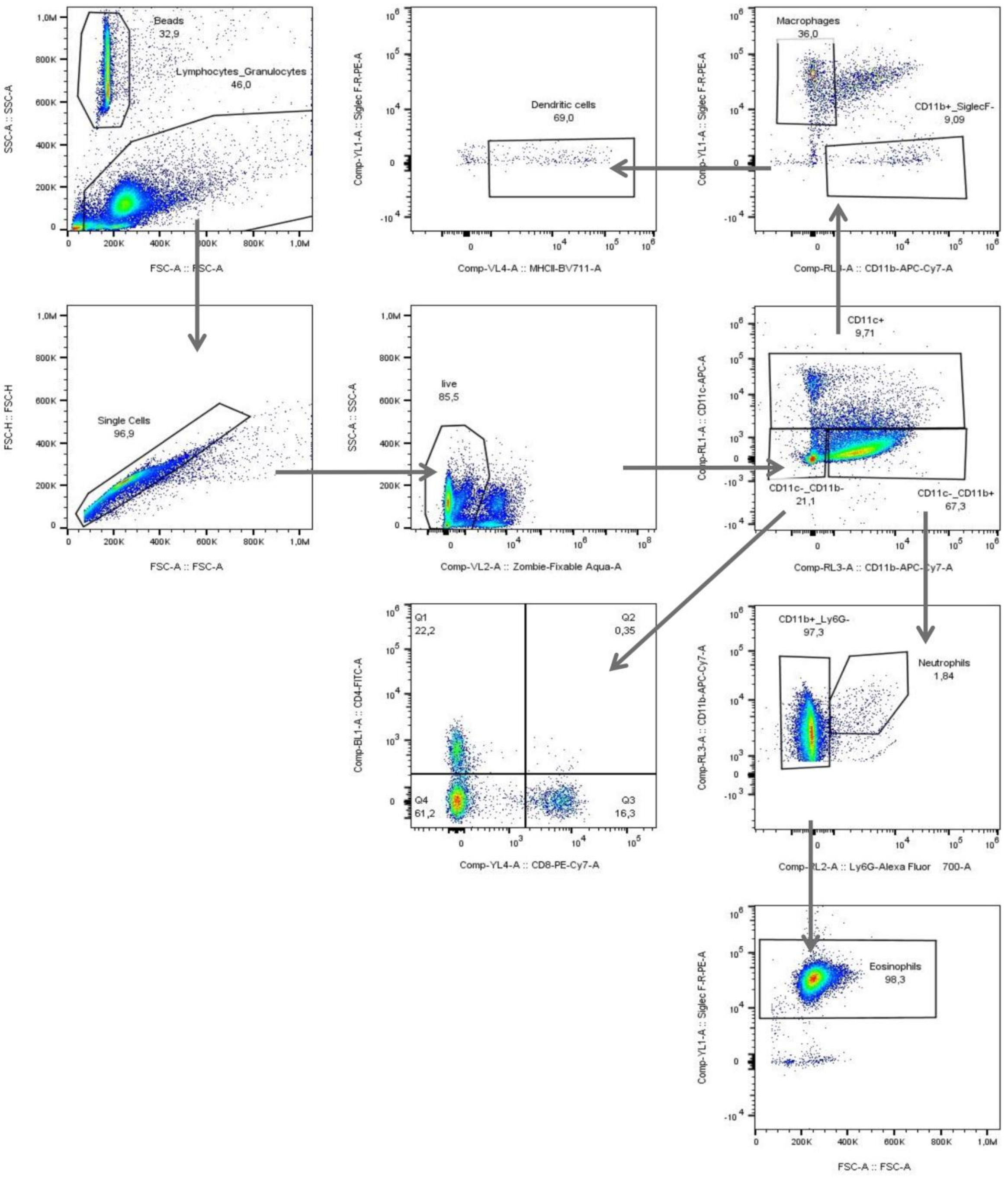

Supplementary figure 1. Gating strategy panel 1 (BAL analysis). After the exclusion of doublets and dead cells, cells were divided into CD11c^{+}, CD11b^{+}/CD11c^{-} and CD11b^{-}/CD11c^{-} cells. CD11c^{+} cells were further divided into macrophages (gated as CD11c^{+}/Siglec F^{+}) and Siglec F^{-} cells which were then gated for dendritic cells (CD11c^{+}/Siglec F^{-}/MHCII^{+}) using an MHCII marker. CD11b^{+}/CD11c^{-} cells were further gated using the Ly6G and Siglec F markers. Neutrophils were identified as CD11b^{+}/CD11c^{-}/Ly6G^{+} cells, eosinophils as CD11b^{+}/CD11c^{-}/Ly6G^{-}/Siglec F^{+} cells. CD4^{+} T helper cells were gated as CD11b^{-}/CD11c^{-}/CD4^{+} and CD8^{+} T cells as CD11b^{-}/CD11c^{-}/CD8^{+}

cells. Displayed here are representative gatings for an OVA/sal control mouse (A) and an OVA/OVA mouse (B).

(A)

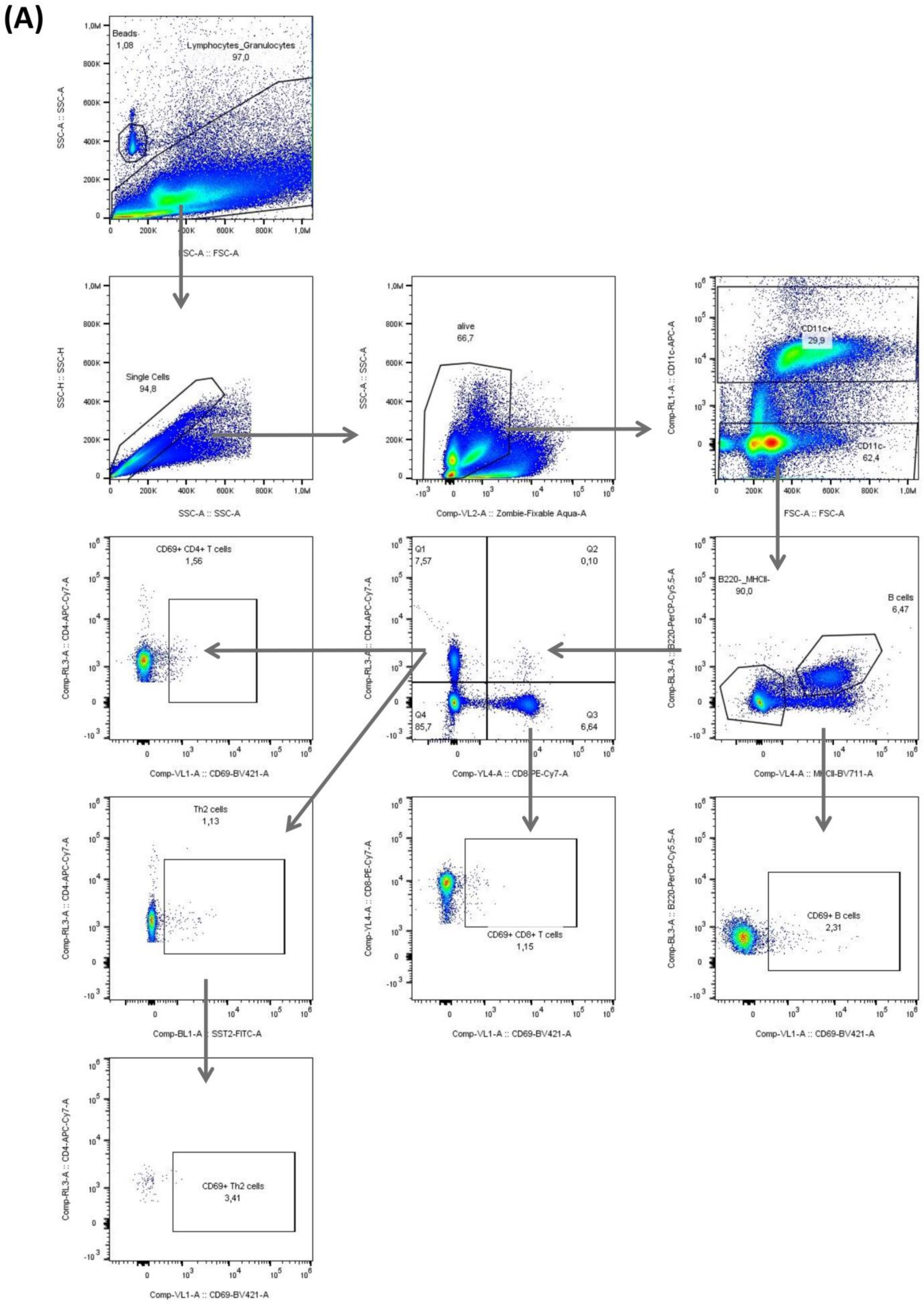
Beads
1,08
Lymphocytes_Granulocytes
97,0
SSC-A :: SSC-A
FSC-A :: FSC-A
Single Cells
94,8
SSC-H :: SSC-H
SSC-A :: SSC-A
alive
66,7
Comp-VL2-A :: Zombie-Fixable Aqua-A
CD11c+
29,9
CD11c-
62,4
Comp-RL1-A :: CD11c-APC-A
FSC-A :: FSC-A
CD69+ CD4+ T cells
1,56
Comp-RL3-A :: CD4-APC-Cy7-A
Comp-VL1-A :: CD69-BV421-A
Q1
7,57
Q2
0,10
Q3
6,64
Q4
85,7
Comp-YL4-A :: CD8-PE-Cy7-A
B220-_MHCII-
90,0
B cells
6,47
Comp-BL3-A :: B220-PerCP-Cy5.5-A
Comp-VL4-A :: MHCII-BV711-A
Th2 cells
1,13
Comp-BL1-A :: SST2-FITC-A
CD69+ CD8+ T cells
1,15
Comp-YL4-A :: CD8-PE-Cy7-A
CD69+ B cells
2,31
CD69+ Th2 cells
3,41

(B)

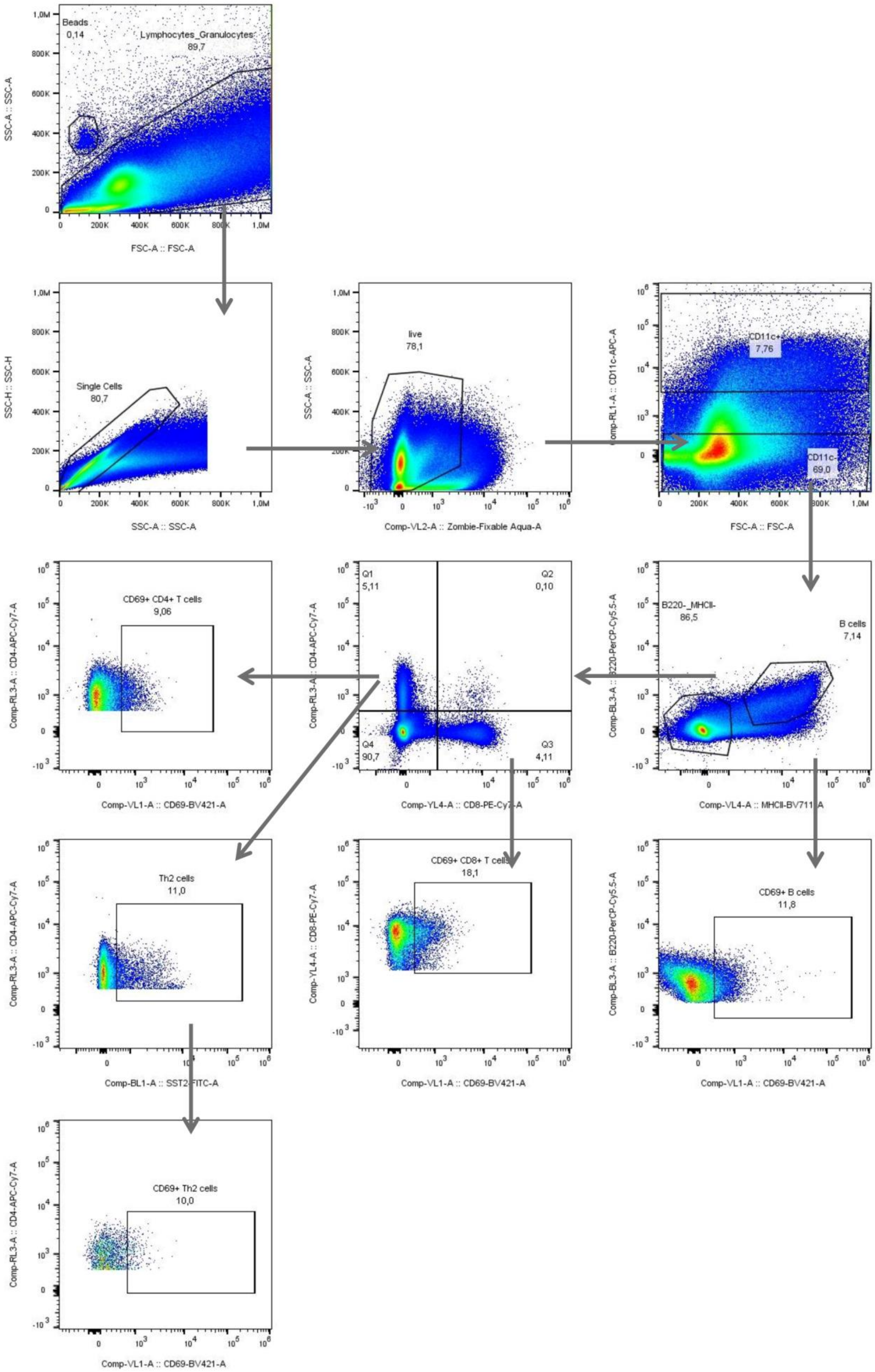
Beads
0,14
Lymphocytes_Granulocytes
89,7
SSC-A :: SSC-A
FSC-A :: FSC-A
Single Cells
80,7
SSC-H :: SSC-H
SSC-A :: SSC-A
live
78,1
SSC-A :: SSC-A
Comp-VL2-A :: Zombie-Fixable Aqua-A
CD11c+
7,76
CD11c-
69,0
Comp-RL1-A :: CD11c-APC-A
FSC-A :: FSC-A
CD69+ CD4+ T cells
9,06
Comp-RL3-A :: CD4-APC-Cy7-A
Comp-VL1-A :: CD69-BV421-A
Q1
5,11
Q2
0,10
Q4
90,7
Q3
4,11
Comp-RL3-A :: CD4-APC-Cy7-A
Comp-YL4-A :: CD8-PE-Cy7-A
B220-_MHCII-
86,5
B cells
7,14
Comp-BL3-A :: B220-PerCP-Cy5.5-A
Comp-VL4-A :: MHCII-BV711-A
Th2 cells
11,0
Comp-RL3-A :: CD4-APC-Cy7-A
Comp-BL1-A :: SST2-FITC-A
CD69+ CD8+ T cells
18,1
Comp-YL4-A :: CD8-PE-Cy7-A
Comp-VL1-A :: CD69-BV421-A
CD69+ B cells
11,8
Comp-BL3-A :: B220-PerCP-Cy5.5-A
Comp-VL1-A :: CD69-BV421-A
CD69+ Th2 cells
10,0
Comp-RL3-A :: CD4-APC-Cy7-A
Comp-VL1-A :: CD69-BV421-A

Supplementary figure 2. Gating strategy panel 2. After the exclusion of doublets and dead cells, cells were gated for $CD11c^{+}$ and $CD11c^{-}$ cells. The latter were further gated by using B220 and MHCII markers. B cells were gated as $CD11c^{-}/B220^{+}/MHCII^{+}$. $CD11c^{-}/B220^{-}/MHCII^{-}$ cells were further gated for $CD4^{+}$ T cells ($CD11c^{-}/B220^{-}/MHCII^{-}/CD4^{+}$ and $CD8^{+}$ T cells ($CD11c^{-}/B220^{-}/MHCII^{-}/CD8^{+}$). Th2 cells were identified as $ST2^{+}$ $CD4^{+}$ T cells. The activations status of different lymphocyte subsets was determined using the CD69 marker. Displayed here are representative gatings of an OVA/sal control mouse (A) and an OVA/OVA mouse (B).

(A)

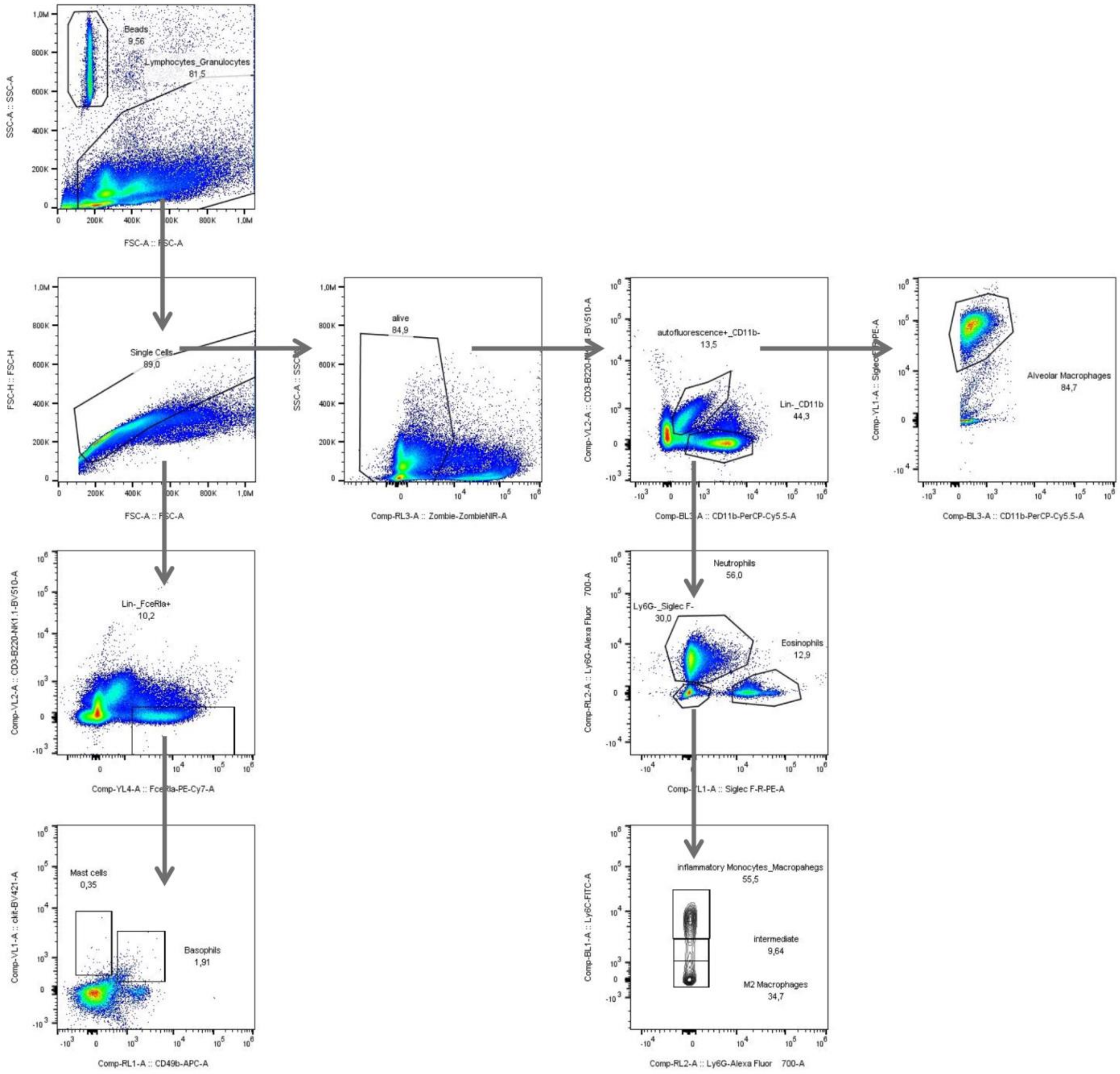
Beads
9,56
Lymphocytes_Granulocytes
81,5
SSC-A :: SSC-A
FSC-A :: FSC-A
Single Cells
89,0
FSC-H :: FSC-H
alive
84,9
Comp-RL3-A :: Zombie-ZombieNIR-A
autofluorescence+_CD11b-
13,5
Lin-_CD11b
44,3
Comp-BL3-A :: CD11b-PerCP-Cy5.5-A
Alveolar Macrophages
84,7
Comp-BL3-A :: CD11b-PerCP-Cy5.5-A
Lin-_FceRIa+
10,2
Comp-VL2-A :: CD3-B220-NK1.1-BV510-A
Comp-YL4-A :: FceRIa-PE-Cy7-A
Neutrophils
56,0
Ly6G-_Siglec F-
30,0
Eosinophils
12,9
Comp-YL1-A :: Siglec F-R-PE-A
Mast cells
0,35
Basophils
1,91
Comp-VL1-A :: ckit-BV421-A
Comp-RL1-A :: CD49b-APC-A
inflammatory Monocytes_Macropahegs
55,5
intermediate
9,64
M2 Macrophages
34,7
Comp-BL1-A :: Ly6C-FITC-A
Comp-RL2-A :: Ly6G-Alexa Fluor 700-A

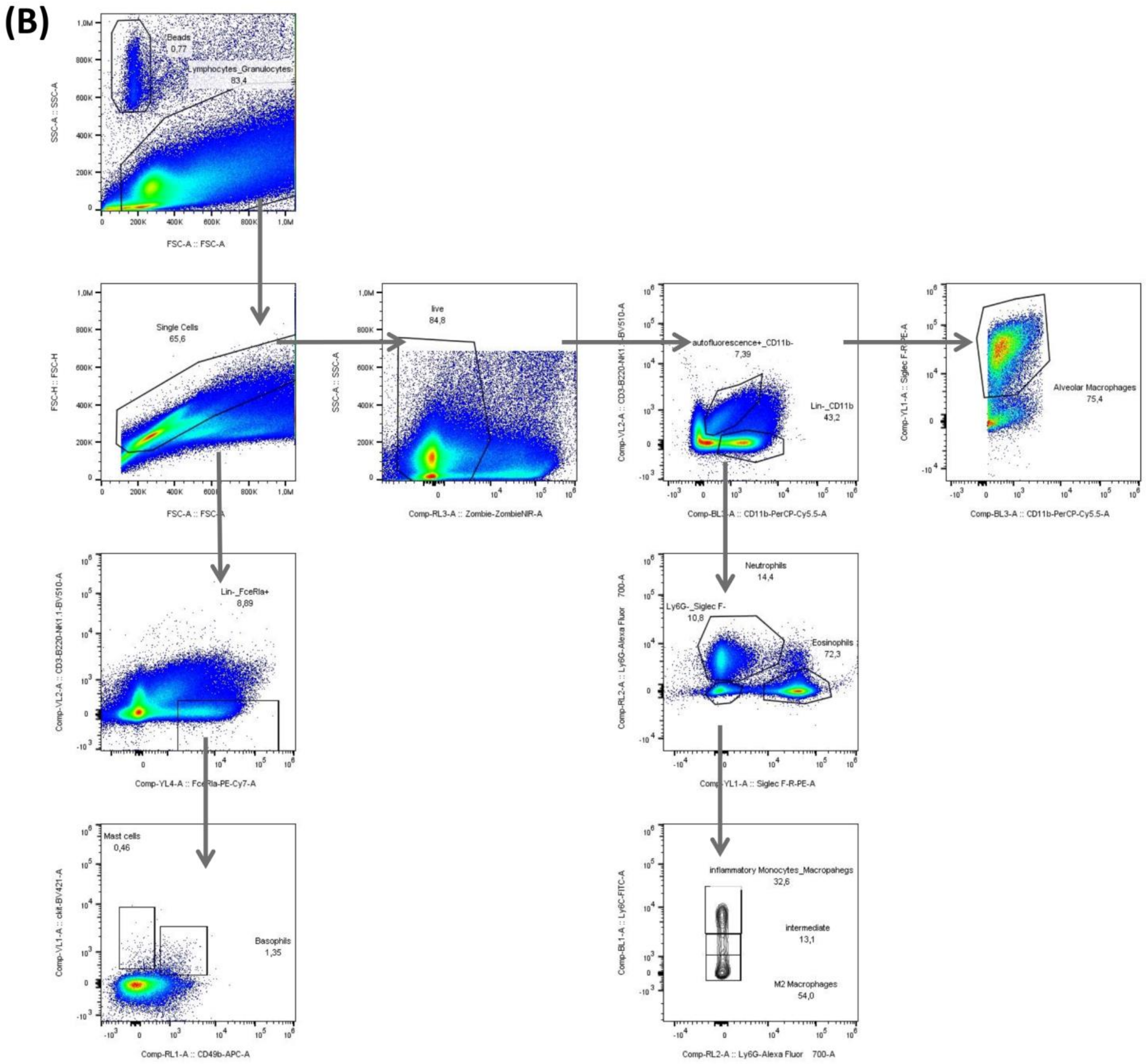

Supplementary figure 3. Gating strategy panel 3. After the exclusion of doublets and dead cells, alveolar macrophages were gated as autofluorescence^{+}/CD11b^{-}/Siglec F^{+} cells. CD3^{-}/NK1.1^{-}/B220^{-}/CD11b^{+} cells were further gated for neutrophils (CD3^{-}/NK1.1^{-}/B220^{-}/CD11b^{+}/Ly6G^{+}/Siglec F^{-}) and eosinophils (CD3^{-}/NK1.1^{-}/B220^{-}/CD11b^{+}/Ly6G^{-}/Siglec F^{+}). CD3^{-}/NK1.1^{-}/B220^{-}/CD11b^{+}/Ly6G^{-}/Siglec F^{-} cells were subdivided into inflammatory monocytes/macrophages (CD3^{-}/NK1.1^{-}/B220^{-}/CD11b^{+}/ Ly6G^{-}/Siglec F^{-}/Ly6C^{high}) and M2-polarized macrophages (CD3^{-}/NK1.1^{-}/B220^{-}/CD11b^{+}/Ly6G^{-}/Siglec F^{-}/Ly6C^{low}) by using the Ly6C marker. Mast cells and basophils were gated without prior dead cell exclusion. Mast cells were gated as FcεRIα^{+}/CD117^{+}/CD49^{-} and basophils as FcεRIα^{+}/CD117^{+}/CD49^{+}. Displayed here are representative gatings for an OVA/sal control mouse (A) and an OVA/OVA mouse (B).

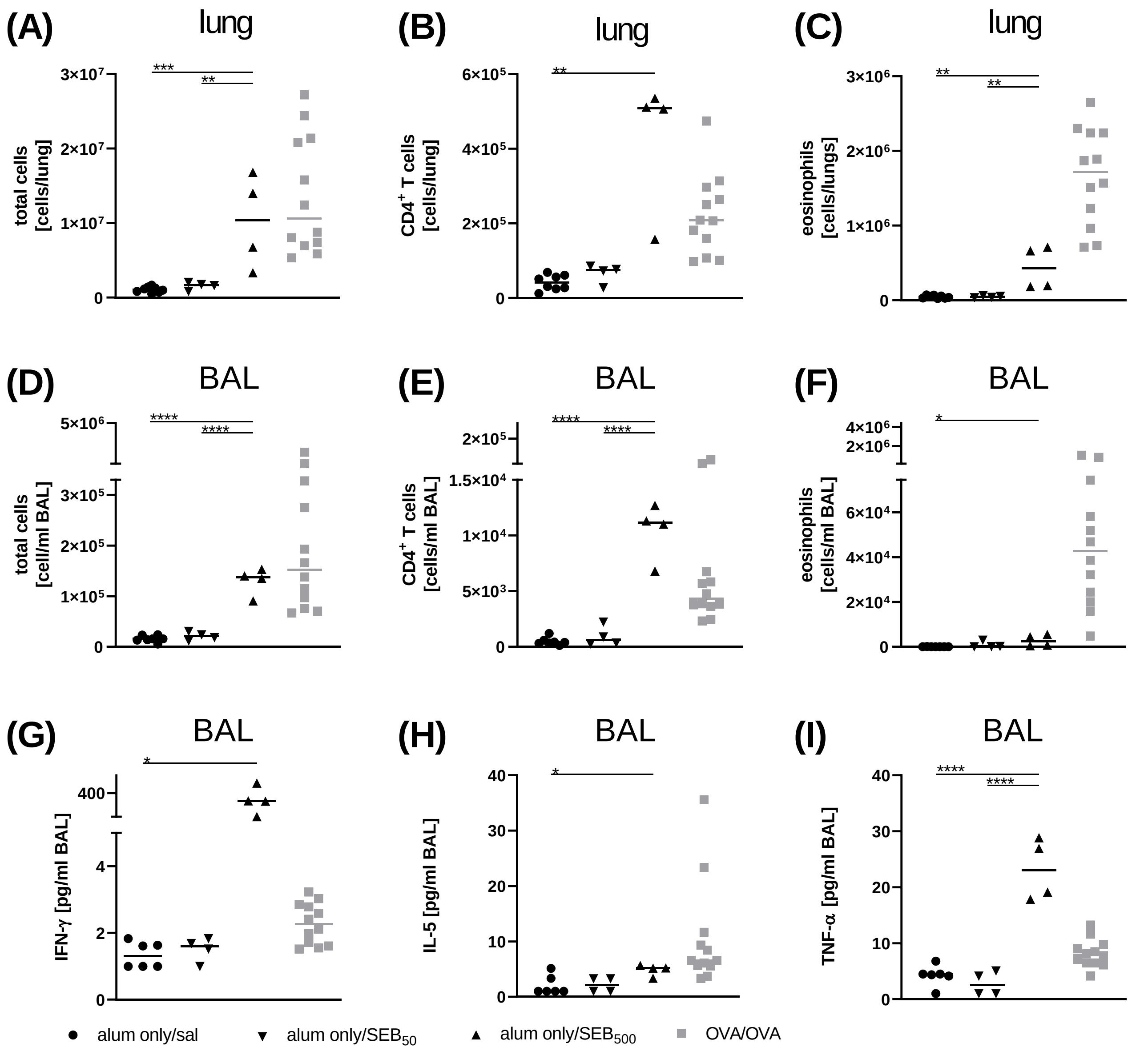

Supplementary figure 4. Intranasal treatment with different *Staphylococcus aureus* enterotoxin B doses leads to cell recruitment to the respiratory tract and a local cytokine response 48 hours after the last application. Mice were mock-sensitized with alum only in weekly intervals (d 0, 7, 14). One week after the last mock-sensitization mice were either intranasally (i.n.) treated with PBS (control (●): alum only/sal) or with 50 ng or 500 ng of the *S. aureus* enterotoxin B (SEB) (50 ng (▼): alum only/SEB_{50}; 500 ng (▲): alum only/SEB_{50}) on three consecutive days (d 21, 22, 23). For the induction of allergic airway inflammation (shown as a reference) mice were sensitized three times with 10 µg ovalbumin (OVA) in 1 mg aluminium hydroxide in weekly intervals (d 0, 7, 14) and one week later i.n. challenged thrice with 100 µg OVA (d21, 22, 23) (AAI (■): OVA/OVA). On day 25 (48h after the last treatment), leukocytes from lungs and bronchoalveolar lavage (BAL) were flow-cytometrically analyzed with respect to the total cell count ((A) lung, (D) BAL), absolute numbers of $CD4^+$ T cells ((B) lung, (E) BAL) and eosinophils ((C) lung, (F) BAL). Concentrations of IFN-γ (G), IL-5 (H) and TNF-α (I) were measured in BAL. Data are compiled from two independent experiments and show individual mice and the median. Groups ●, ▼, and ▲ were compared by one-

way ANOVA with Bonferroni post-hoc test or Kruskal-Wallis test with Dunn's post-hoc test depending on whether data were normally distributed or not (according to Shapiro-Wilk testing). *$p < 0.05$, ** $p < 0.01$, *** $p < 0.005$, **** $p < 0.0001$.

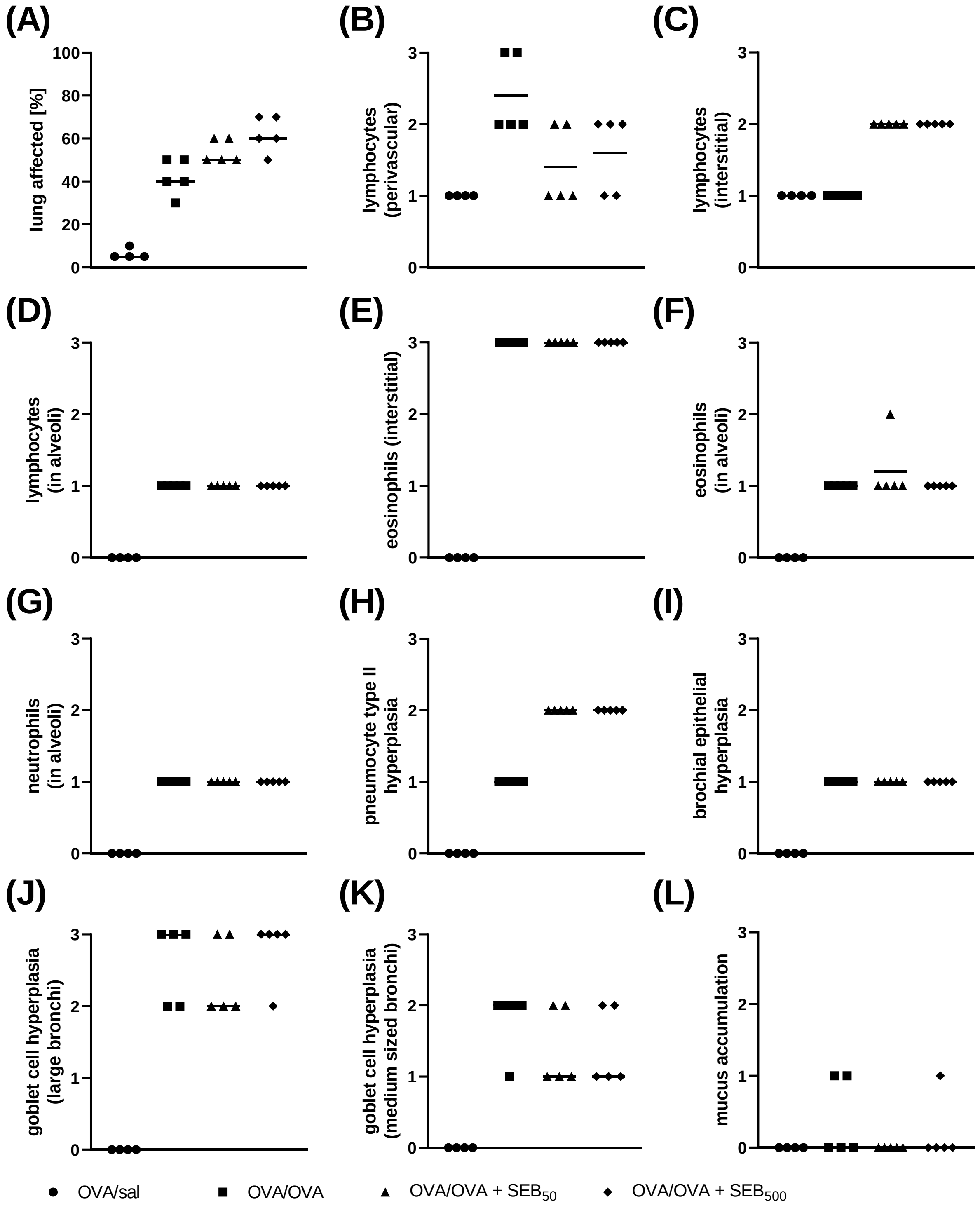

Supplementary figure 5. Histopathological analysis of lungs after the induction of AAI alone or of AAI with i.n. SEB-treatment during the allergic challenge. Mice were OVA-sensitized and challenged with PBS (controls, OVA/sal), challenged with OVA (OVA/OVA) or challenged with OVA together with 50 ng (OVA/OVA + SEB_{50}) or 500 ng SEB (OVA/OVA + SEB_{500}). Histopathological analyses were performed as described. The % lung affected (A), scores from H&E-staining (B - I) and from PAS-staining (J - L) are shown for individual mice.

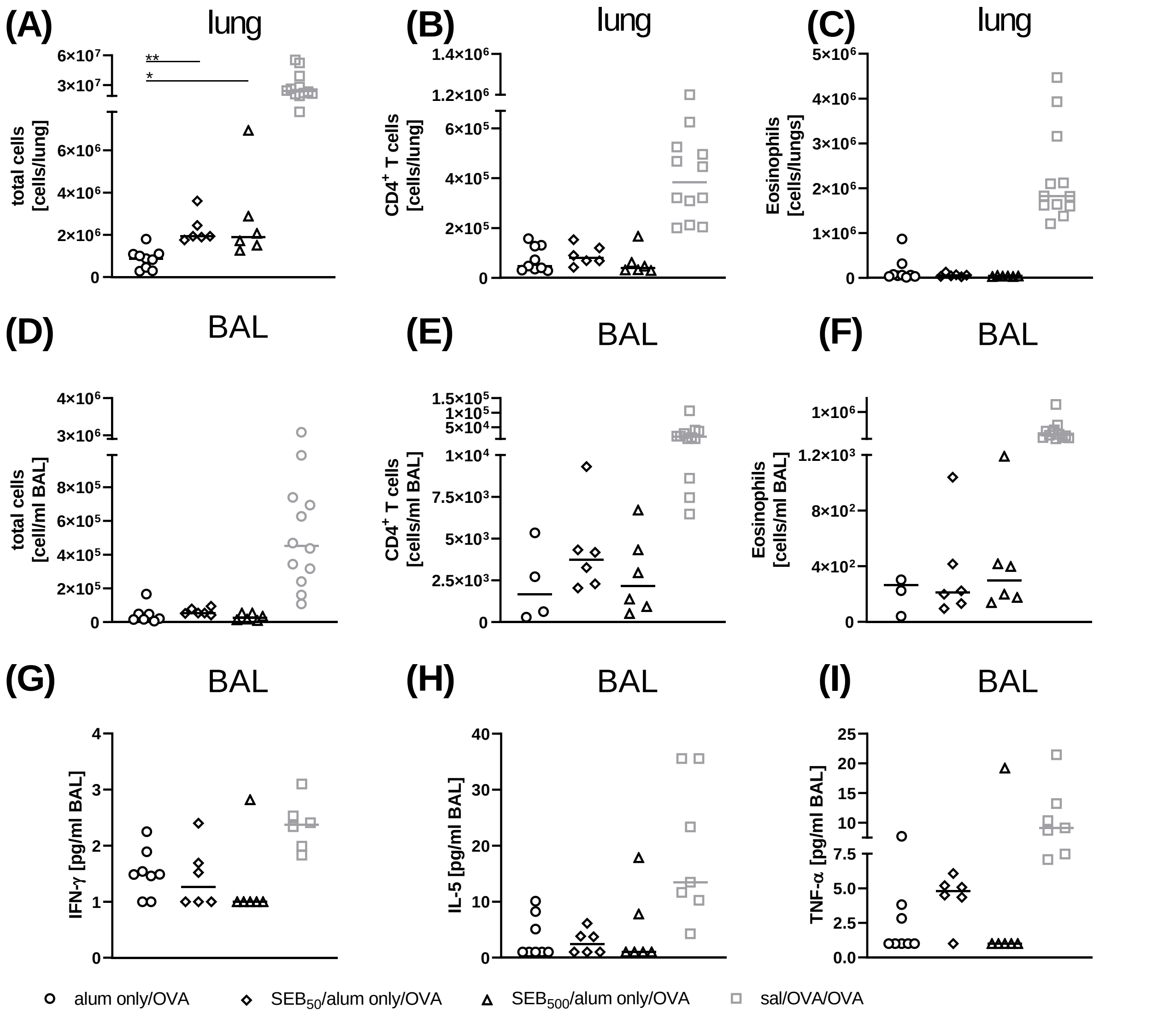

Supplementary figure 6. Intranasal treatment with different *Staphylococcus aureus* enterotoxin B doses leads to a long term increase in the total cell number in the lungs and bronchoalveolar lavage of treated mice. To examine long term effects of i.n. treatment with the different doses of *S. aureus* enterotoxin B (SEB) alone, mice were treated i.n. with either 50 ng (◇; SEB_{50}/alum only/OVA) or 500 ng (△; SEB_{500}/alum only/OVA) SEB on three consecutive days (d -3, -2, -1), intraperitoneally (i.p.) injected with alum only in weekly intervals (d 0, 7, 14) and challenged i.n. three times with OVA (d 21, 22, 23) one week later. The control group (○; alum only/OVA) was not treated with SEB, was i.p. injected with alum only in weekly intervals (d 0, 7, 14) and also challenged i.n. three times with OVA (d 21, 22, 23) one week later. For the induction of allergic airway inflammation alone (shown as a reference), mice were treated i.n. with PBS on three consecutive days (d -3, -2, -1) and were three times sensitized i.p. with 10 µg ovalbumin (OVA) in 1 mg alum in weekly intervals (d 0, 7, 14) and challenged i.n. three times with OVA (d 21, 22, 23) one week later (□; sal/OVA/OVA). On day 25, leukocytes from lungs and bronchoalveolar lavage (BAL) were flow-cytometrically analyzed with respect to the total cell count ((A) lung, (D) BAL),

absolute numbers of $CD4^+$ T cells ((B) lung, (E) BAL) and eosinophils ((C) lung, (F) BAL). Concentrations of IFN-γ (G), IL-5 (H) and TNF-α (I) were measured in BAL. Data are compiled from at least two independent experiments for each group and are shown for individual mice with the group median. Groups ○, ◇, and △ were compared by one-way ANOVA with Bonferroni post-hoc test or Kruskal-Wallis test with Dunn's post-hoc test depending on whether data were normally distributed or not (according to Shapiro-Wilk testing). *$p < 0.05$, ** $p < 0.01$.
